# IKKβ in Myeloid Cells Controls the Host Response to Lethal and Sublethal *Francisella tularensis* LVS Infection

**DOI:** 10.1371/journal.pone.0054124

**Published:** 2013-01-22

**Authors:** Sylvia Samaniego, Kenneth B. Marcu

**Affiliations:** 1 Graduate Program in Genetics, Stony Brook University, Stony Brook, New York, United States of America; 2 Biochemistry and Cell Biology Department, Stony Brook University, Stony Brook, New York, United States of America; University of Iowa Carver College of Medicine, United States of America

## Abstract

**Background:**

The NF-κB activating kinases, IKKα and IKKβ, are key regulators of inflammation and immunity in response to infection by a variety of pathogens. Both IKKα and IKKβ have been reported to modulate either pro- or anti- inflammatory programs, which may be specific to the infectious organism or the target tissue. Here, we analyzed the requirements for the IKKs in myeloid cells *in vivo* in response to *Francisella tularensis* Live Vaccine Strain (*Ft.* LVS) infection.

**Methods and Principal Findings:**

In contrast to prior reports in which conditional deletion of IKKβ in the myeloid lineage promoted survival and conferred resistance to an *in vivo* group B streptococcus infection, we show that mice with a comparable conditional deletion (IKKβ cKO) succumb more rapidly to lethal *Ft.* LVS infection and are unable to control bacterial growth at sublethal doses. Flow cytometry analysis of hepatic non-parenchymal cells from infected mice reveals that IKKβ inhibits M1 classical macrophage activation two days post infection, which has the collateral effect of suppressing IFN-γ^+^ CD8^+^ T cells. Despite this early enhanced inflammation, IKKβ cKO mice are unable to control infection; and this coincides with a shift toward M2a polarized macrophages. In comparison, we find that myeloid IKKα is dispensable for survival and bacterial control. However, both IKKα and IKKβ have effects on hepatic granuloma development. IKKα cKO mice develop fewer, but well-contained granulomas that accumulate excess necrotic cells after 9 days of infection; while IKKβ cKO mice develop numerous micro-granulomas that are less well contained.

**Conclusions:**

Taken together our findings reveal that unlike IKKα, IKKβ has multiple, contrasting roles in this bacterial infection model by acting in an anti-inflammatory capacity at early times towards sublethal *Ft.* LVS infection; but in spite of this, macrophage IKKβ is also a critical effector for host survival and efficient pathogen clearance.

## Introduction

NF-κB is an important signaling pathway for the induction and regulation of innate and adaptive immune responses toward bacterial infection. Microbial components and pro-inflammatory stress-like signals universally impact the activation of NF-κB transcription factors via the inhibitor of NF-κB kinase (IKK) signalosome complex. The signalosome contains two catalytic kinases, IKKα and IKKβ, and a third docking/regulatory subunit, IKKγ/NEMO [1]. The catalytic IKKs mediate the phosphorylation and subsequent degradation of the IκB family of cytoplasmic inhibitory proteins. IκB degradation liberates NF-κB transcription factors, resulting in nuclear translocation and target gene activation (for reviews see [2], [3], [4]). Canonical NF-κB activation requires IKKβ and IKKγ for IκB degradation, while the role of IKKα in the canonical signalosome is less clear. In addition, IKKα and IKKβ have also been shown to possess a variety of NF-κB-independent functions by regulating effectors of cell cycling, apoptosis, specific cellular differentiation pathways, chromatin activity and inflammatory responses {reviewed in. [5], [6], [7], [8]).

In addition to the well-established roles the IKKs have on the induction of inflammation and adaptive immune responses, myeloid IKKα and IKKβ also limit inflammation in response to the extracellular bacterium Group B Streptococcus (GBS) or by *E. coli* LPS- (lipopolysaccharide) induced septic shock [9], [10], [11], [12]. This unexpected behavior of the IKKs in myeloid cells led us to further investigate their anti-inflammatory properties in the context of the intracellular bacterium *Francisella tularensis* (*Ft*.).

*Francisella tularensis* is a highly infectious, Gram-negative, intracellular bacterium and is the causative agent of tularemia. Due to the high virulence of the human pathogenic strain ShuS4, the attenuated Live Vaccine Strain (*Ft*. LVS) is commonly used in mouse models of infection due to its ability to approximate human disease [13], [14]. *Ft*. infects several cell types [15], [16], [17], [18], [19], [20], but macrophages are considered the primary target of early infection [19], [21], [22]. Bacteria disseminate to colonize the lungs, liver and spleen presumably through the hematogenous route [13], [23]. Examination of *Ft*. LVS or ShuS4 infected organs indicates that lung, liver and spleen each contribute distinct immune reactions [15], [24], [25], [26]. Within 72 hours of intradermal (i.d.) or intraperitoneal (i.p.) inoculation of *Ft*. LVS, a pro inflammatory gene expression profile is evident in liver and to a lesser extent in spleen, while lungs respond in an anti inflammatory manner [24].

The virulence of *Francisella* has been attributed to its ability to down-modulate and/or evade host defenses. For example, outside the cell, *Francisella* are opsonized with complement proteins but are able to resist killing by complement-mediated lysis [27], [28], [29]. Opsonized bacteria are taken up readily by phagocytic cells, but avoid degradation by preventing fusion of host phagosomes to lysosomes and subsequently escape to the cytosol where they replicate [30], [31], [32], [33]. Although *Ft*. is a Gram-negative bacterium, it has evolved an unusual LPS structure, which only minimally activates the pattern recognition receptor TLR4 (Toll-like Receptor-4) [34], [35], [36], and instead, innate immune recognition of *Ft*. LVS is mediated through TLR2 (Toll-like receptor-2) [37], [38], [39], [40].

*Francisella* also interferes with anti-microbial defenses and inflammatory signaling pathways in several cell types, including macrophages and neutrophils. For example, *Ft*. LVS can prevent anti-microbial ROS (reactive oxygen species) production and NADPH oxidase assembly in human neutrophils [18]. *Ft*. LVS actively down-modulates TNF-α and IL-1 in the J774A murine macrophage cell line; this coincides with the inhibition of IκBα degradation and cross-tolerization to *E. coli* LPS (a TLR4 agonist) resulting in interference of NF-κB signaling [41], [42].

There is also evidence that *Ft.* LVS affects macrophage polarization [43]. As early as 24 hours post infection, infected peritoneal elicited macrophages show properties of anti-inflammatory, alternatively-activated/M2 polarization resulting in increased expression of the M2 markers: mannose receptor (CD206), Fizz-1, Arg-1 and Ym1 [43]. Similar to the T cell polarization paradigm, macrophage polarization also depends on cytokine cues induced by the local environment (reviewed in [44], [45]). In agreement with this, *Francisella* interferes with IFN-γ signaling, a key cytokine involved in macrophage polarization and bacterial control. *Ft.* LVS and its closely related subspecies *Ft. novicida* modulate IFN-γ signaling by suppressing tyrosine phosphorylation of the STAT1 transcription factor (Signal Transducer and Activator of Transcription 1) in human and murine mononuclear cells and this correlates with up-regulation of the STAT1 inhibitor SOCS3 (suppressor of cytokine signaling) [46].

Since infection with *Ft*. suppresses key inflammatory regulators, we hypothesized that the anti-inflammatory properties of myeloid IKK may compound and/or contribute to the progression of tularemia *in vivo*. To investigate this, we used myeloid-specific conditional knockout mice (IKK cKO) [47] to assess the roles of the IKKs in an intradermal (i.d.) model of *Ft*. LVS infection. We chose the liver as the model organ for our study because during tularemic infection, it supports pro-inflammatory changes [24], is colonized early in infection and has an abundant source of macrophages [48].

We found that myeloid IKKβ is required for survival during septic challenge, while IKKα cKO mice displayed mortality rates comparable to control mice. In a sublethal model of infection, flow cytometry anaylsis of hepatic non-parenchymal cells showed that loss of myeloid IKKβ, but not IKKα, results in polarization toward M1 macrophages in the liver early in the course of infection. Interestingly we found these effects are transient, as by mid-infection, macrophages polarize towards the M2a lineage. Loss of myeloid IKKβ also induces protracted elevations in IFN-γ expressing CD8^+^ T cells, which persist throughout the course of infection. Despite these changes, IKKβ cKO mice are defective in control of bacterial growth. Finally, histological analysis shows that infection results in abnormal granulomatous liver responses in both strains of IKK cKO mice.

## Methods

### Animals

Myeloid specific *ikk* deletions were generated by crossing IKKα or IKKβ floxed mice with the LysM Cre expressing mouse strain [49] to generate IKKα *^flox/flox^*-LysM Cre or IKKβ *^flox/flox^*-LysM Cre strains, all of which were maintained on a C57BL/6 genetic background, as previously described [47]. To simplify nomenclature used throughout this text, mice are herein referred to as IKK*^f/f^* to represent control IKK floxed strains without myeloid deletion and IKKα cKO or IKKβ cKO to represent corresponding myeloid *ikk* conditional deletions. Mice were housed in a facility equipped with a 12∶12 hour light:dark cycle in ventilated cages; and were fed a normal chow diet and autoclaved water ad libitum.

### Ethics Statement

All procedures were performed in strict accordance with State University of New York at Stony Brook IACUC approved protocol (Permit Number 0163).

### Bacteria

*Ft*. LVS (ATCC 29684, American Type Culture Collection, Manassas, VA) were a kind gift from the laboratory of Dr. Martha Furie, and were grown according to published protocols [50]. Briefly, frozen stocks were streaked on chocolate II agar supplemented with hemoglobin and IsoVitaleX (BD Biosciences, San Jose, CA) and grown at 37°C for 3 days. Single colonies were inoculated into pre-warmed (37°C, 5% CO2) Mueller Hinton-II broth and incubated at 37°C with shaking for 16–18 h. Bacteria were used at OD_600_ between 0.2–0.4.

### Infection Model

Bacteria were serially diluted to the indicated doses in sterile PBS in a final volume of 100 µl. Eight- to twenty-week old female mice were shaved and injected intradermally (i.d.) at the base of the tail. Mice were monitored for signs of illness (hunched posture, ruffled fur, loss of appetite, etc.). At lethal doses, these symptoms sometimes did not appear until hours before succumbing to infection. In addition, occasionally mice that appeared ill had spontaneously recovered. Sublethal infection did not induce symptoms of illness. Doses, listed as colony forming units (CFU), were confirmed by a retroplate colony count assay as described below.

### Survival Analysis

The 50% Lethal Dose (LD_50_), was determined using the IKK*^f/f^* parental strain. Mice were infected i.d. with 10^6^, 10^7^ or 10^8^ CFU of *Ft*. LVS and monitored daily for survival.

For comparison of survival between all strains, mice (10–15 per group) were injected with an LD_50_ dose 10^8^ CFU i.d. and monitored twice daily over a period of 14 days. Kaplan-Meier survival curves were generated and relative mortalities in response to lethal infection were compared by Mantel-Cox log-rank test.

### Organ Burden Determination and Retroplate Colony Counts

Organ burden was determined using limiting-dilution culture. Briefly, sections of the median lobe (∼0.1–0.2 g) of infected livers were aseptically removed and placed in a pre-weighed sterile tube containing 1 ml of sterile PBS. The remaining liver and section were weighed. The section was homogenized in a stomacher bag (Fisher Scientific, Pittsburgh, PA) and serially diluted. Neat homogenates and serial dilutions were plated and incubated for 3 days at 37°C. The resulting colonies were counted and represented as a concentration per gram of liver. This concentration was then multiplied by the weight of the total liver and corresponding dilution factor to derive the total organ burden. Retroplate colony counts were performed in a similar manner. First, serial dilutions on the inoculation dose were made. Dilutions were then plated and incubated as above. The resulting colonies were counted, multiplied to respective dilution factors and represented as CFU/ml. The limit of detection for these assays was 200 CFU.

### Histology

Organs were fixed in 10% neutral buffered formalin (Sigma Diagnotics, St. Louis, MO) and embedded in paraffin, sectioned at 5 µm rehydrated to water through graded alcohols. Tissues were stained with hematoxylin and eosin (H & E), dehydrated in graded alcohols, cleared with xylene and mounted with Acrymount (EMS, Hatfield, PA). An experienced pathologist performed a blinded analysis on H & E sections to identify any abnormalities in lung, liver and spleen. Images were captured on an Olympus BX41 light microscope (Olympus, Tokyo, Japan).

Granuloma counts from 8 mice/group were quantified by examination of 10 fields/liver under 200× magnification. Poisson distribution was assumed and fitted to Poisson regression by taking the square root of count data and mean comparisons were made with one-way ANOVA and Tukey’s post-test. Data was then back-transformed into original units of granulomas/200× field (0.150 mm^2^).

### Immunohistochemistry

Tissues were fixed in neutral-buffered formalin for 48 hrs, embedded in paraffin and sectioned at 5 µm. Deparaffinization, rehydration and antigen retrieval was performed by incubating slides in Trilogy™ solution (Cell Marque, Rocklin, CA) for 1 hr at 90°C. Slides were rinsed in TBST (Tris Buffered Saline-Tween; Thermo-Scientific, Freemont, CA), blocked in goat serum for 30 minutes at RT, and rinsed again in Tris buffer. Slides were incubated with polyclonal anti-*Ft.* LVS rabbit serum or anti-iNOS (Abcam, Cambridge, MA) primary antibody (diluted 1∶50 in Tris buffer) for 16 hours at 4°C. Slides were rinsed and then incubated with AP-conjugated goat anti-rabbit secondary antibody (diluted 1∶100; Invitrogen, Carlsbad, CA) and developed using Vulcan Fast Red Chromagen (Biocare Medical, Concord, CA) for 10 minutes. To counterstain, slides were rinsed in H_2_O and dipped twice in Mayer’s Hematoxylin (Cancer Diagnostics, Morrisville, NC). Slides prepared for the detection of cleaved caspase-3 (CC-3) were processed in similar manner with a few modifications. Endogenous peroxidase was inhibited using Peroxidazed-1 blocking reagent (Biocare Medical) for 5 minutes prior to blocking in goat serum. CC-3 primary antibody (Cell Signaling Technology, Danvers, MA) was diluted 1∶50 and applied as above. A secondary goat anti-rabbit antibody (1∶500 dilution; supplied with Vectastain, ABC kit, Vector Laboratories, Burlington, CA) was applied for 30 minutes. Slides were rinsed in Tris buffer and ABC reagent from the same kit was added for 30 minutes. ImmPACT™ DAB solution (Vector Labs) was applied for 6 minutes, rinsed and counterstained as above. Staining was absent in control slides where primary antibody was omitted. Occasional weak background was noted in uninfected controls. Images of immunostained slides were captured using a 20× objective on an Aperio ScanScope CS slide scanner (Aperio,Vista, CA ) to create whole-slide digital images. Images were subsequently analyzed using Aperio ImageScope v11.12.2.760 Positive-Pixel Count Algorithm v9 (Aperio) for systematic identification of *Ft*. antigen, iNOS or cleaved caspase-3 positivity. The software samples the image and reports a staining intensity (a value proportional to the amount of transmitted light) and generates a pseudo-colored markup image to verify staining intensities. Staining intensities are thus reported as negative (255, blue), weak positive (220, yellow), positive (175, orange) and strong positive (100, brown). Using these parameters, we defined weak positive (yellow) as non-specific/background staining, while positive and strong positive values (orange+red/brown) were considered to be specific staining. The algorithm was applied to both positive and negative control tissues to ensure validity of the settings.

Positive granulomas were counted by examination of 10 fields/liver under 200× magnification. Poisson distribution was assumed and fitted to Poisson regression by taking the square root of count data and mean comparisons were made with one-way ANOVA and Tukey’s post-test. Data are represented as percent positive granulomas.

### Non-parenchymal Cell (NPC) Isolation

NPC’s were isolated by the methods of Rasmussen *et al* [51]. Briefly, mice were injected with 10^6^ CFU of *Ft*. LVS and euthanized at the indicated time points. On the day of harvest, livers were perfused with 1 mM Citrate (Sigma, St. Louis, MO) in Hanks Buffered Saline Solution (HBSS, Gibco/Invitrogen Carlsbad, CA). Livers were minced to 1 mm pieces and placed in digest buffer containing 0.05% Collagenase II, 0.002% DNAse1 and 1% BSA (all from Sigma-Aldrich, St. Louis, MO) in HBSS, rotating at 75 RPM, 37°C for 45 minutes. Digests were triturated 10X and filtered through a 70 µm cell strainer (BD Falcon, San Jose, CA). Cells were pelleted and resuspended in ACK hypotonic buffer (0.15 M NH_4_Cl, 10 mM KHCO_3_, 0.1 mM Na_2_EDTA) for 5 minutes to lyse RBCs. Cells were washed in PBS and NPCs were isolated by flotation method using Optiprep density gradient (AxisShield, Oslo, Norway), according to manufacturer protocol C-24. Upper and lower isolates were collected and the NPC fraction was confirmed by flow cytometry. The upper isolate was >97% CD45+ (eBioscieinces, San Diego, CA).

### Flow Cytometry

NPCs isolated from infected livers were stained with antibodies for surface and/or intracellular markers for immunophenotyping and cytokine positivity according to manufacturer suggested protocols. Briefly, harvested cells were immediately incubated in growth media (RPMI 1640, 5% heat inactivated FBS (Invitrogen/Life Technologies, Carlsbad, CA) without antibiotics) containing 1 µg/ml GolgiPlug (BD Biosciences, San Jose, CA) for 6 hours to prevent cytokine release; no exogenous stimuli were added during the incubation period. GolgiPlug was added to all subsequent flow cytometry buffers until the fixation step. Cells were washed 1X in PBS and 1X in FACS Stain Buffer (FSB: 1% heat inactivated FBS, 0.09% w/v NaN_3_ in PBS). Fcγ receptors were blocked with CD16/32 antibody (eBiosciences, San Diego, CA). Surface epitopes were stained with antibody for 30 min., washed 2 times with FSB. Cells were fixed and permeabilized using BD Cytofix/Cytoperm buffer kit (BD Biosciences, San Jose, CA) for 20 min. Intracellular antibodies were added to cells and incubated for 30 min. Unlabeled antibodies required secondary labeling was carried out in a third staining step as above. All labeling and permeabilization steps took place in the dark with rotation. The following antibodies were used in this study: Arg-1 (Everest Biotech, Oxfordshire, UK), PE-CD3α (B111922, BioLegend, San Diego, CA), FITC-CD3ε (145-2C11, eBiosciences, San Diego, CA), FITC-CD4 (RM4-5, BioLegend), FITC-CD8α (53-6.7, BioLegend), PE-CD11b (M1-70, BioLegend), PerCP-Cy5.5-CD11b (M1-70, BD Biosciences, San Jose, CA), FITC-CD45 (30.F11, BioLegend), APC-F4/80 (BM8, eBiosciences), FITC-F4/80 (BM8, eBiosciences), *Ft*. LVS (Rabbit polyclonal, a generous gift of Dr. Jorge Benach), APC-IFN-γ (XMG1.2, BD Biosciences), PE-IL-10 (JES5-16E3, BD Biosciences), APC-IL-12p40/70 (C15.6, BD Biosciences), FITC-pan-Neutrophils/Ly-6B.2 (7/4, AbD Serotec, Raleigh, NC), PerCP-Cy5.5-NK1.1 (PK136, BD Biosciences), RELMα/FIZZ1 (Rabbit Polyclonal, Abcam, Cambridge, MA), PE-Donkey anti rabbit IgG (Abcam), PE-Donkey anti goat IgG (Abcam). Acquisition was performed on FacsCalibur flow cytometer (BD Biosciences, San Jose, CA) and data was analyzed using FlowJo V9.0.1, (TreeStar, Ashland, OR). Cell counts are normalized as counts per gram of liver.

### Statistical Analysis

Data were analyzed by one-way ANOVA with Tukey’s multiple comparison post-test using GraphPad Prism 6 software (GraphPad Software, Inc., La Jolla, CA).

## Results

### Reduced Survival of Myeloid IKKβ-deficient Mice Following Lethal Intradermal *Ft.* LVS Challenge

The IKKs are important signaling factors involved in orchestrating host immune responses to a wide variety of pathogens, yet the requirements for IKK signaling in myeloid cells during *Ft.* LVS infection are unknown. To address this, we employed Cre/Lox mediated recombination in which IKKα or IKKβ are conditionally deleted from the myeloid compartment of adult mice [47], [49], [52].

The severity of *Francisella* infection varies by bacterial strain, host genetic background, dose and route of administration [13], [23], [53]. In order to establish the correct dosage for our model system, we began with a pilot experiment in which we intradermally (i.d.) injected IKK*^f/f^* mice at various doses of *Ft*. LVS in order to establish the median lethal dose (LD_50_). After retroplate assay correction, the LD_50_ was determined to be 10^8^ CFU of *Ft*. LVS (Fig. S1 A–C).

**Figure 1:**
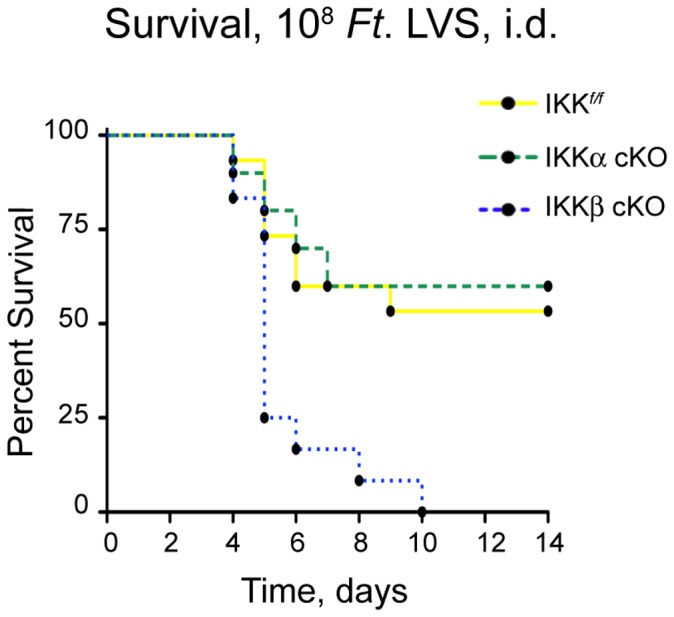
Myeloid IKKβ is essential for host resistance to a lethal, i.d. *Ft.* LVS infection. IKK*^f/f^*, IKKα cKO and IKKβ cKO mice were injected i.d. with the LD_50_ dose of 10^8^ CFU of *Ft*. LVS and monitored for survival by the Kaplan-Meier method (n = 10–15 mice per group; Log-rank P value = 0.0014).

We first investigated if either IKKα or IKKβ in myeloid cells were required for host survival to *Ft*. LVS infection. We injected IKK*^f/f^*, IKKα cKO and IKKβ cKO mice with the LD_50_ dose of 10^8^ CFU *Ft.* LVS and scored animals for their relative survival rates. All IKKβ cKO mice succumbed to infection by day 10 with a comparative Log-rank test P value of 0.0014 (Fig. 1); there were no significant differences in the survival rates among IKK*^f/f^* and IKKα cKO mice.

### Both IKKα and IKKβ in Myeloid Cells Contribute to Granulomatous Response in the Liver during Sublethal *Ft*. LVS Infection

Next we asked how conditional loss of either IKK in myeloid cells affects disease progression using a sublethal model of infection. In order to determine the sublethal dose, we tested mice at lower doses of inoculum (Fig. S1 B–C). IKK*^f/f^* and IKKα cKO mice had an 80% survival rate at 10^7^ CFU of *Ft*. LVS, in contrast IKKβ cKO mice showed increased sensitivity to infection resulting in a 60% survival rate at this same dose. All three strains of mice survived i.d. infection of 10^6^ CFU of *Ft*. LVS for a period of 90 days; and thus we used this dose for all sublethal experiments.

Infection with *Ft*. LVS results in dissemination to lung, liver and spleen, and results in inflammatory infiltration and/or development of granuloma-like foci in target organs. We examined these tissues by hematoxylin and eosin (H & E) staining in a time course experiment to assess histopathological changes.

**Figure 2:**
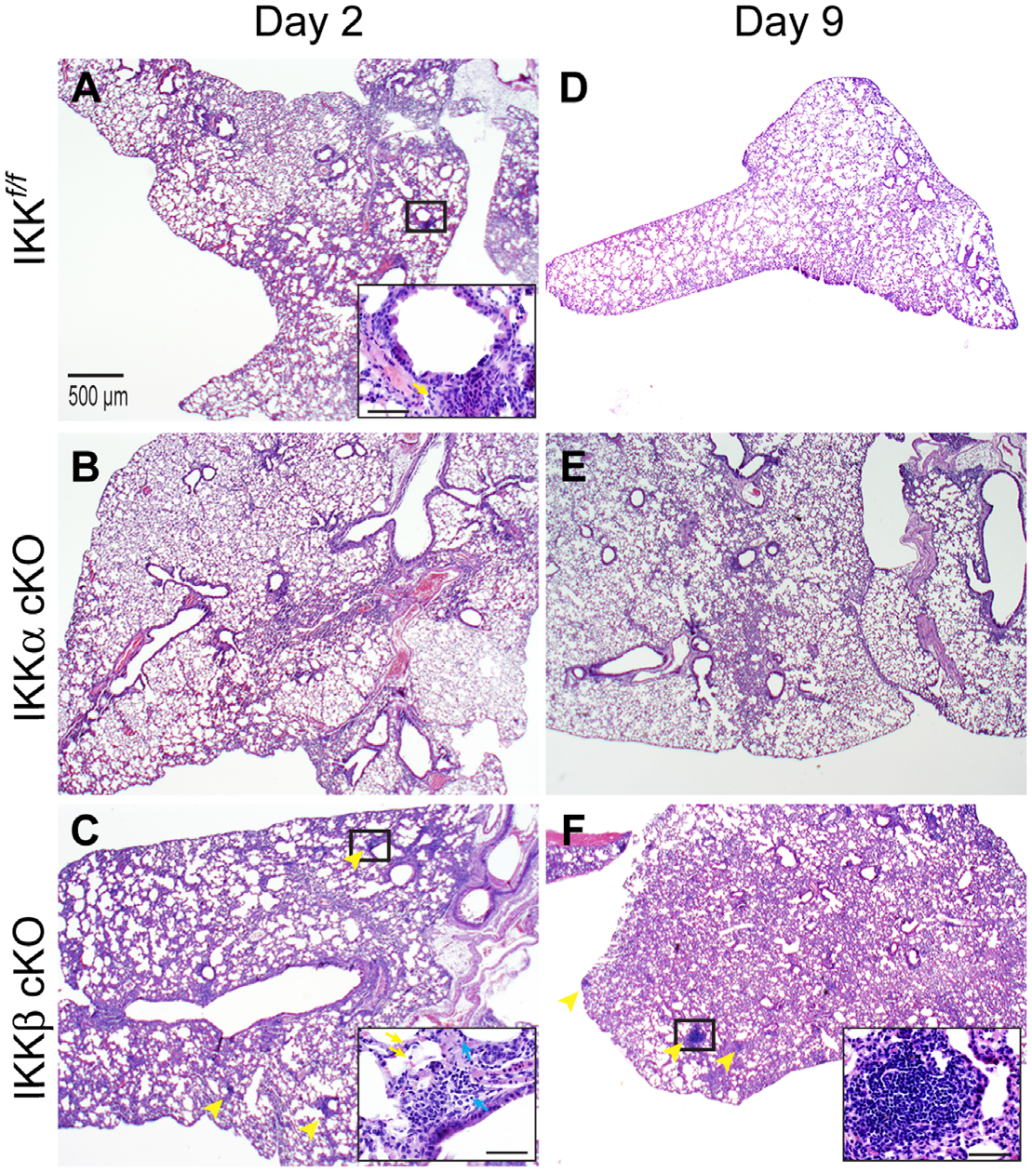
Dissemination of *Ft*. LVS leads to lymphocytic aggregate development in IKKβ cKO mouse lung. H & E stained lung tissue was examined for histological changes at days 2 and 9 (**A–F**) after i.d. infection with 10^6^ CFU of *Ft*. LVS. (**A**) IKK*^f/f^* and (**B**) IKKα cKO mice show evolution and (**D, E**) resolution of inflammation, while (**C and F**) IKKβ cKO mice exhibit a delay in resolution concordant with the appearance of (**F, inset**) lymphocytic aggregates. Figure insets are taken from regions marked by rectangles. Yellow arrows indicate neutrophils, blue arrows indicate lymphocytic infiltrate, yellow arrowheads indicate lymphocytic aggregates. Scale bar = 500 µm, 20× magnification, inset scale bar = 50 µm, 400× magnification). Representative sections are shown from at least three independent experiments.

Lung histology was normal in all three strains of mice prior to infection (data not shown). At two days post-infection, mild lung reactions were observed (Fig. 2A–C and Fig. S2). Peribronchial inflammatory infiltrates were sometimes present and consisted largely mononuclear cells and a few neutrophils (Fig. 2A and C, insets). Areas of peribronchial infiltrate were more numerous in IKKβ cKO mice and these infiltrates also contained lymphocytes (Fig. 2C, inset). Alternatively, lungs from IKKα cKO mice showed the lowest overall degree of inflammation (Fig. 2B) at this same time point. Progressing through days 6 (data not shown) and 9 (Fig. 2D–F and S2), all three strains of mice showed some degree of thickening in the alveolar walls and septae. Luminal alveolar exudate and dilated capillaries were sometimes observed, indicating possible onset of a mild interstitial pneumonia. We noted, on occasion, a minor degree of activation in cells of the mesothelial lining, a symptom of visceral pleuritis. However, only small areas were affected and this did not occur with any regularity. Interestingly, lungs from IKKβ cKO infected mice developed multiple areas of organized lymphocytic aggregates (Fig. 2F), while this was a rare occurrence in IKK*^f/f^* or IKKα cKO mice. These aggregates resemble inducible bronchial-associated lymphoid tissue (iBALT), which can be induced in humans and mice upon exposure to infection and inflammation and are considered tertiary sites of B and T cell priming reactions [54]. Immunohistochemical analysis to detect *Ft*. LVS antigen (Fig. S2) showed a limited infection within the lung parenchyma. The overall conclusion about lung reactions in all three strains of mice was that they were relatively mild. These findings are consistent with previous reports for intradermal infection, not only with *Ft*. LVS [55], but also with the more virulent strain *Francisella tularensis* Schu4 [23].

**Figure 3:**
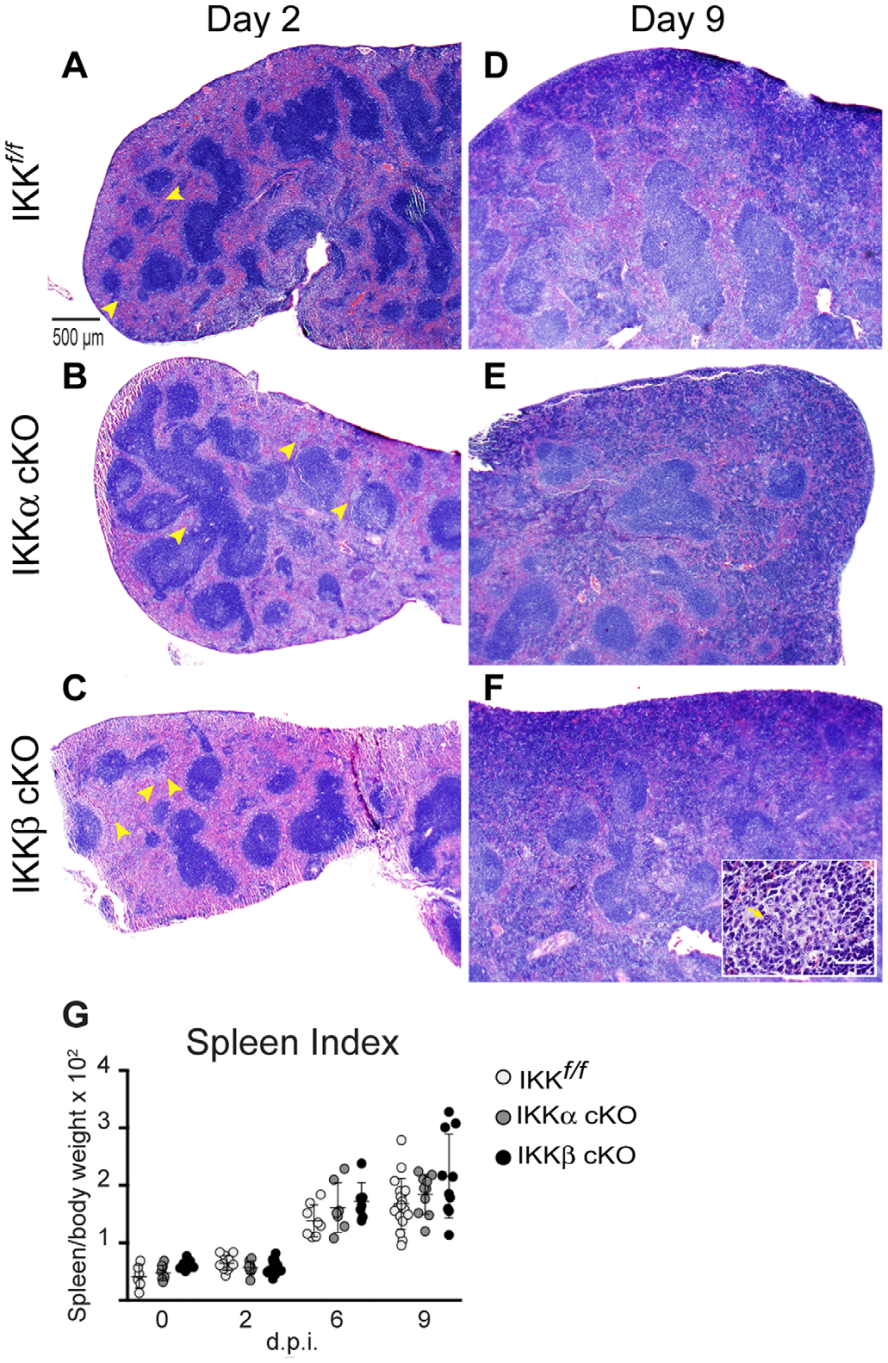
Exacerbated histopathology in the spleen in IKKβ cKO mice. Spleen sections were stained with H & E and evaluated for histopathological changes at days 2 and 9 post i.d. infection with 10^6^ CFU of *Ft*. LVS. Granuloma foci developed in the red pulp of the spleen (yellow arrowheads) in (**A**) IKK*^f/f^*, (**B**) IKKα cKO and (**C**) IKKβ cKO mice as early as 2 days post-infection. (**D–F**) These foci increased in number by day 9. Inset in (**F**) is representative of a typical granuloma within the red pulp. The yellow arrow points to a neutrophil within the granuloma. (**G**) All strains of mice developed splenomegaly during the course of infection as determined by the spleen index (ratio of spleen weight to body weight×100). Scale bar for panels A–F = 500 µm, 20× magnification, inset scale bar in F = 50 µm, 400×magnification. Representative sections are shown from at least three independent experiments.

The spleen and liver undergo pronounced inflammatory reactions upon *Ft.* LVS infection and are characterized by the development of granuloma foci, which act as a microenvironment that serves to contain infection and clear debris from infected and dying cells [19], [23], [51], [56]. Histological examination of the spleens from uninfected mice appeared normal and showed clearly demarcated red and white pulp. Follicles, germinal centers and the surrounding cuff of the marginal zone were well represented (data not shown). At two days post infection, the red pulp was marginally expanded, but other structures were qualitatively normal in all three strains. A few small granuloma-like lesions began to appear within the red pulp of most animals (Figs. 3A–C and Fig. S3). More severe histopathological changes to splenic architecture progressed through days 6 and 9 post-infection (data not shown and Fig. 3D–F, respectively). The white pulp showed evidence of expansion and macrophage and lymphocytic infiltrates were seen in the red pulp. The number of granulomas marginally increased in IKK*^f/f^* and IKKα cKO mice. However, in IKKβ cKO infected mice, the red pulp was often completely effaced with coalescing granulomas making accurate quantification difficult. Neutrophils were sometimes present within these granulomas (Fig. 3F, inset). Splenomegaly was apparent in all strains (Fig. 3G). Comparisons of spleen/total mouse weight ratios (spleen index) showed a ∼3 fold change by day six. The spleen index modestly increased to 3.5–4 fold by day nine. No significant differences in spleen indices between strains were observed. Immunohistochemical analysis of *Ft*. antigen in spleen at 2 days post infection showed numerous bacterial foci (Fig. S3 A–C) in all three strains of mice. Bacteria were localized predominantly in the red pulp, but were occasionally observed in the white pulp as well (Fig. S3 A). By day 9, there was a marked decrease in the number of bacterial foci, indicating resolution of infection.

**Figure 4:**
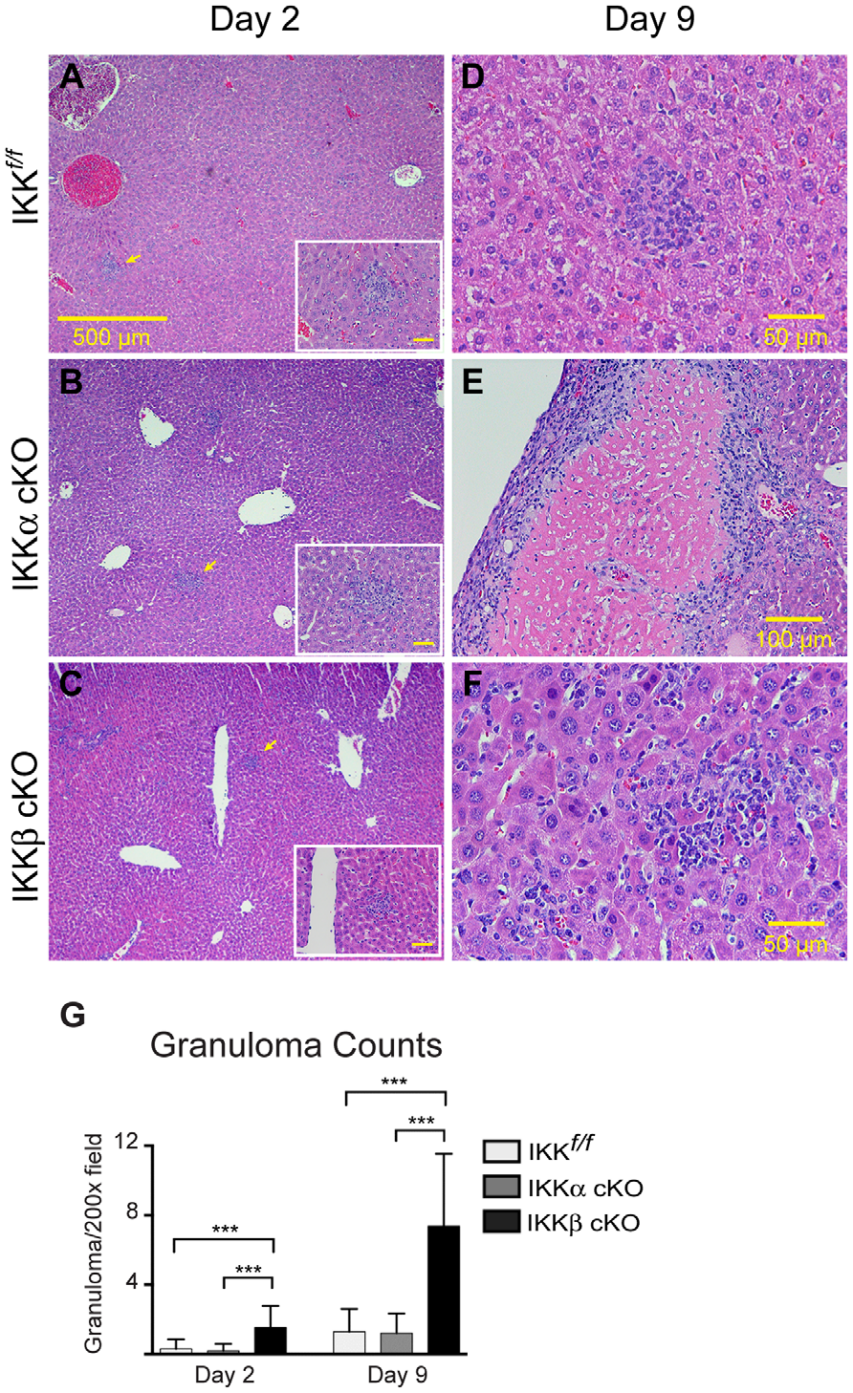
Loss of either IKK kinase results in defects in hepatic granuloma development. Comparison of early and late granuloma development after sublethal i.d. infection with 10^6^ CFU of *Ft.* LVS. Representative H & E stained liver sections are shown for (**A**) IKK*^f/f^*, (**B**) IKKα cKO and (**C**) IKKβ cKO infected mice at two days post-infection. Granulomas, indicated by yellow arrows (40× magnification, scale bars = 500 µm), are magnified in the inset of each panel (400× magnification, scale bars = 30 µm). Panels (**D–F**) are representative granulomas at 9 days post-infection (panels D and F are 200× magnification, scale bar = 50 µm; Panel E is 100×magnification, scale bar = 100 µm). (**G**) Granulomas were quantified as counts per 200× field and were analyzed by one-way ANOVA and Tukey’s ad hoc post-test. Data are pooled from two independent experiments (each with n = 4 mice per group) for a total of n = 8 mice per group; ***P<0.0001.

Granuloma formation in liver was evident at two days post-infection in all three strains (Fig. 4A–C). At day 2, granuloma counts in IKKβ cKO mice were at least two fold greater in number than those of IKK*^f/f^* or IKKα cKO mice (Fig. 4G, P<0.0001). By day 9, granuloma formation continued to increase in IKKβ cKO at a rate far greater than either IKK*^f/f^* or IKKα cKO mice. Additionally, the granuloma-like structures that developed in IKKβ cKO infected mice were often extremely small and decondensed, resulting in livers replete with inflammatory cells (Fig. 4F). Some granulomas in IKKα cKO mice developed into very large, macroscopically discernable structures that contained a central core filled with necrotic and cellular debris (Fig. 4E; note that panel 4E is a lower magnification than 4D and 4F), and although large, these structures were well contained by a cuff of mononuclear cells, epitheloid histiocytes and occasional lymphocytes. Although both spleen and liver showed increased numbers of granuloma foci in IKKβ cKO mice, the liver also showed clear discernable defects in granuloma maintenance in both mutant strains of mice. Since the liver reacts with a strong pro-inflammatory response to *Ft*. LVS intradermal infection [24] and contains a rich source of macrophages, we focused the remainder of our experiments on the liver to further investigate the anti-inflammatory effects of myeloid IKKβ during the course of tularemia.

### Myeloid IKKβ is Required for Control of Bacterial Growth in the *Ft*. LVS Infected Liver

**Figure 5:**
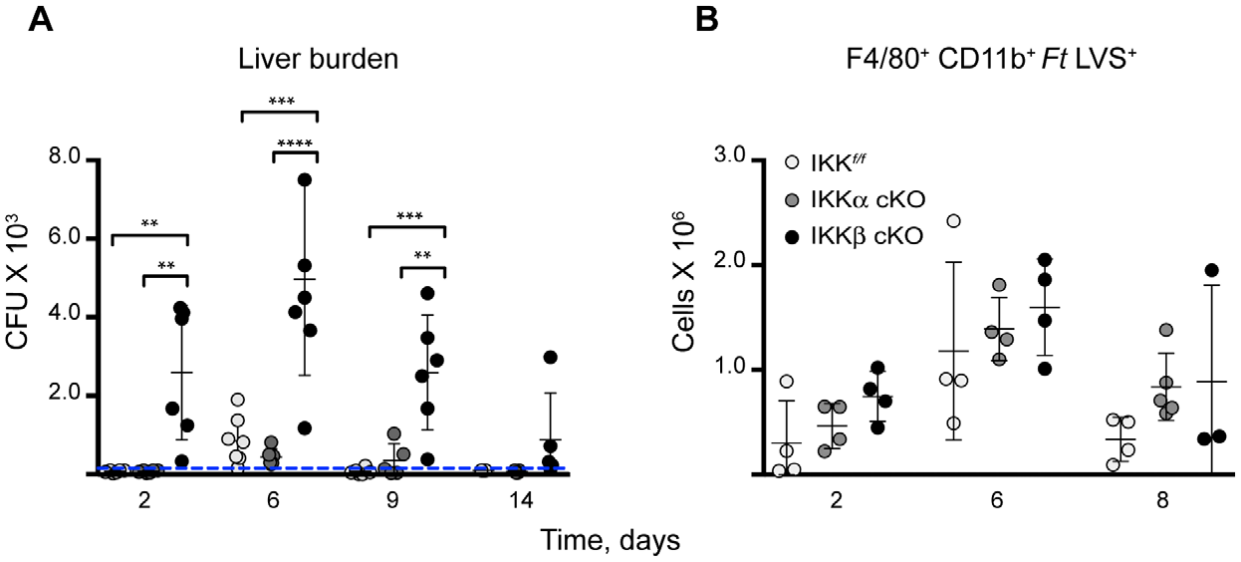
IKKβ controls *Ft*. LVS growth in the liver. (**A**) Organ burdens were determined by retroplate assay from liver homogenates of mice injected i.d. with 10^6^ CFU *Ft.* LVS at days 2, 6, 9, and 14. Data is presented as CFU of *Ft*. LVS per gram of liver. Data were pooled from two independent experiments. The dashed line represents the assay limit of detection, 200 CFU. (n for IKK*^f/f^*, IKKα cKO, IKKβ cKO on day 2 = 8; day 6 = 7; day 9 = 6; day 14 n = 6) (**B**) Liver macrophages were analyzed for infection by flow cytometry. Statistical analysis was performed by one-way ANOVA followed by Tukey’s ad hoc post-test, (n for IKK*^f/f^*, IKKα cKO, IKKβ cKO on day 0∶5/5/5; day 2∶4/4/4; day 6∶4/4/4; day 8∶4/4/3, respectively). Bars represent the mean ± SD. **P<0.01, ***P<0.0005, ****P<0.0001.

Bacterial colonization is first detectable in *Ft*. infected livers at 2–3 days post infection [19], [24], [51], [56]. In order to determine if the observed granuloma defects in the liver were due to increased bacterial colonization, a retroplate time course study to determine organ burden was performed on liver homogenates from mice infected with 10^6 ^CFU of *Ft*. LVS. At day 2 post infection, both IKK*^f/f^* and IKKα cKO mice showed limited colonization of the liver while, even at this early time point, IKKβ cKO mice presented with increased bacterial loads that persisted through day 14 (Fig. 5A).

**Figure 6:**
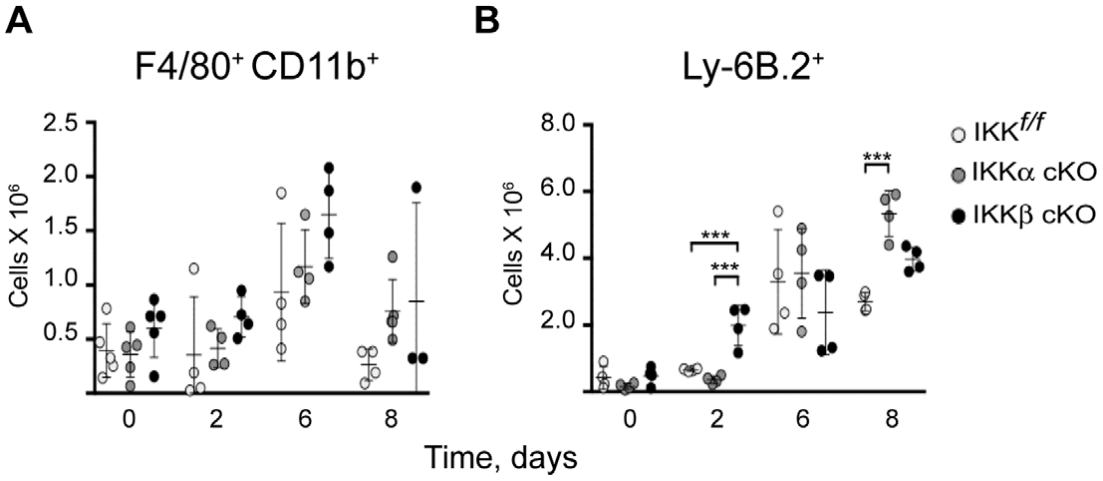
Myeloid lineage response in *Ft*. LVS infected livers. We analyzed (**A**) macrophage and (**B**) neutrophil populations in the liver by flow cytometry during the course of sublethal *Ft*. LVS infection. Statistical analysis was performed by one-way ANOVA followed by Tukey’s ad hoc post-test. (n for IKK*^f/f^*, IKKα cKO, IKKβ cKO on day 0∶5/5/5; day 2∶4/4/4; day 6∶4/4/4; day 8∶4/4/3, respectively). Results are representative of at least two independent experiments. Bars represent the mean ± SD. *P<0.05, **P<0.01, ***P<0.0005, ****P<0.0001.

Macrophages are considered a primary cellular target of *Francisella* infection [22], [57] and are also a major component of *Ft*. induced granulomas [51]. Since macrophages are one of the cell types affected by our conditional knockouts, we next questioned whether the macrophages from these mice were comparably infected relative to control mice. To address this, non-parenchymal cells (NPCs) were isolated from the livers of infected mice, stained with F4/80 and CD11b antibodies followed by intracellular staining for *Ft*. LVS and then subjected to flow cytometry. Interestingly, IKKβ cKO infected mice showed only modest increases in the number of *Ft*. LVS positive macrophages throughout the course of infection (Fig. 5B). To account for a possible loss of cells due to infection, we analyzed macrophage and neutrophil cell populations from each strain. We isolated hepatic NPCs from sublethally *Ft.* LVS infected livers and performed a flow cytometry analysis time course to evaluate the populations of F4/80^+^ CD11b^+^ expressing macrophages and Ly6B.2^+^ expressing neutrophils. Prior to infection (day 0), all three strains of mice yielded comparable numbers of macrophages and neutrophils (Fig. 6A & B, respectively). Modest increases were observed in the macrophage population but persisted throughout the 8-day time course (Fig. 6A). As early as two days post infection, increases in neutrophils were evident in IKKβ cKO mice and by day 8, neutrophils were elevated in both mutant strains, relative to the parental strain (Fig. 6B). Taken together, these data suggest that the increased liver burden in IKKβ cKO mice or granuloma maintenance were due to defects in myeloid function related to control of bacterial growth and spread of infection rather than overt initial increases in macrophage infection (Fig. 5B) or loss of cells from the myeloid compartment (Fig. 6A–B).

### Myeloid Function in the Granuloma

**Figure 7:**
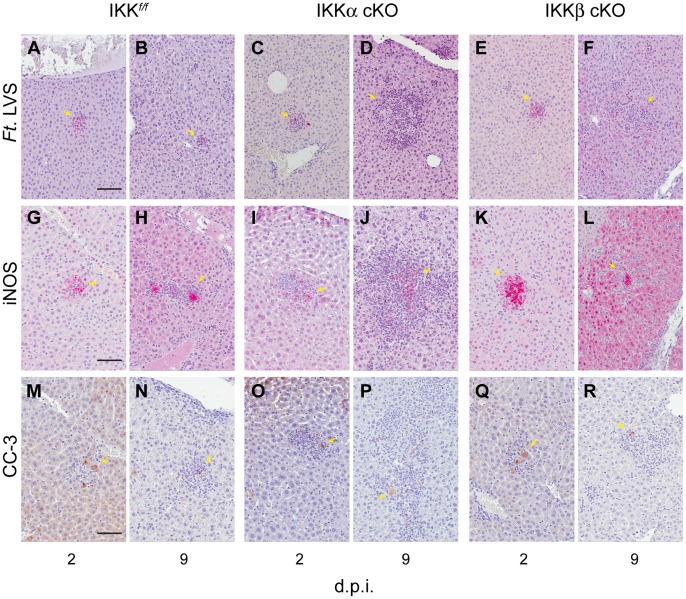
Reactivity of *Ft*. LVS induced granulomas. Mice were challenged i.d. with 10^6^ CFU of *Ft*. LVS and livers were analyzed by immunohistochemistry for anti-*Ft*. LVS antigen, anti-iNOS and anti-cleaved caspase-3 (CC3) at days 2 and 9 post infection. *Ft*. LVS antigens were detected within (**A–B**) IKK*^f/f^*, (**C–D**) IKKα cKO and (**E–F**) IKKβ cKO granulomas at days 2 and 9, respectively. iNOS positivity was determined for (**G–H**) IKK*^f/f^*, (**I–J**) IKKα cKO and (**K-L**) IKKβ cKO mice. Activated caspase-3, an early indicator of apoptosis, was detected in (**M-N**) IKK*^f/f^*, (**O–P**) IKKα cKO and (**Q–R**) in IKKβ cKO granulomas at days 2 and 9 post infection, respectively. 200× magnification, scale bar = 50 µm; d.p.i.: days, post-infection, (n = 4).

To further examine myeloid function in liver granulomas, we performed immunohistochemical assays to detect the spatial localization of LVS antigen, production of the inflammatory mediator iNOS (inducible nitric oxide synthase) and induction of caspase-3 (CC-3) activation. Figure 7 shows representative granulomas from each of the three strains of mice. LVS antigen was largely restricted to the granuloma (Fig. 7. A–F), however, a small number of *Ft.* antigen positive cells (8-11cells/200× field) were sometimes found within the parenchyma. *Ft.* antigen positive cells were almost two-fold higher in IKKβ cKO mice, but this correlated with higher bacterial burdens.

Lindgren *et al* [58] reported that intradermal inoculation of *Ft*. LVS in iNOS (inducible Nitric Oxide Synthase) deficient mice results in decreased host survival, increased bacterial colonization and increased liver damage concurrent with the appearance of numerous small granuloma-like foci in the liver. Moreover, *iNOS*, in addition to induction by IFN-γ, is also a secondary response target gene activated through TLR signal transduction [59], [60]. We next asked if iNOS was similarly expressed in liver granulomas of IKK deficient mice. At two days post infection, we observed iNOS positivity in 54%, 40% and 54% in hepatic granulomas from IKK*^f/f^*, IKKα cKO and IKKβ cKO mice, respectively (Fig. 7 B, H, N). By nine days post-infection (Fig. 7 E, K, Q), only modest increases in the number of iNOS-postive granulomas (∼10%) in IKK*^f/f^* and IKKβ cKO infected mice were noted. In comparison, the number of IKKα cKO iNOS positive granulomas remained essentially unchanged at 42%.

Rapid induction of apoptosis in bacterially infected cells is an immune defense mechanism that helps to limit the spread of infection. We analyzed induction of apoptosis in hepatic granulomas using the apoptotic marker, cleaved caspase-3 (CC-3). CC-3 staining was largely restricted to granulomas. At early time points in infection, ∼35% of all granulomas scored positive CC-3 in all strains of mice. However, only a few (∼1–8) positive cells were found per granuloma (Fig. 7M–R), and this was consistent between all strains of mice. By day nine, CC-3 positive granulomas from IKK*^f/f^* mice was reduced to 11.3%, while IKKα and IKKβ cKO mice retained an overall 25 and 22% CC3 positive granuloma score, respectively.

### IKKβ Inhibits Classical/M1 Macrophage Activation in the Liver in Response to *Ft*. LVS Infection

**Figure 8:**
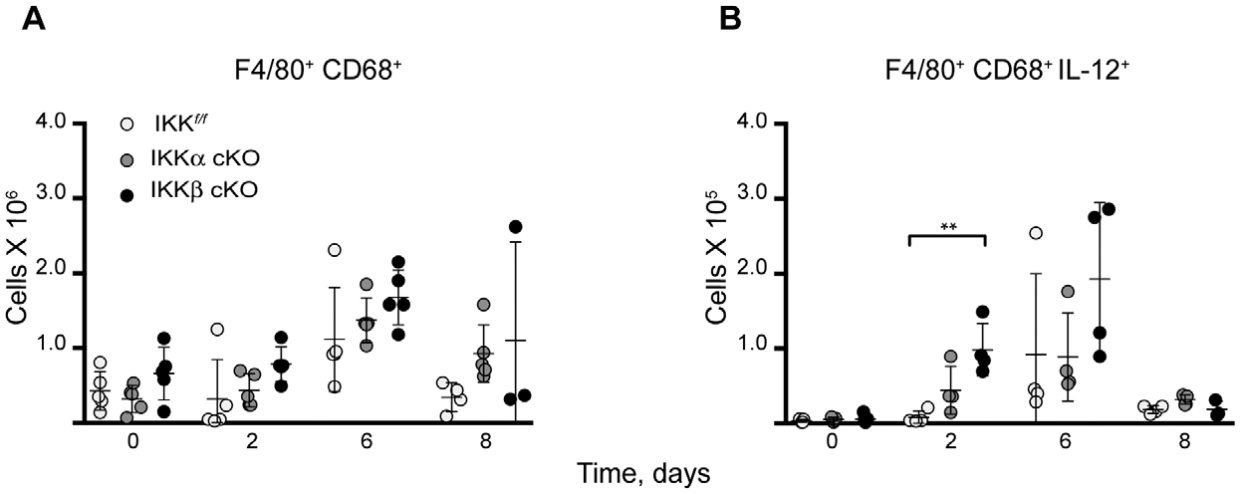
Loss of IKKβ induces temporal M1 activation in response to *Ft.* LVS. Hepatic NPCs were isolated from IKK*^f/f^*, IKKα and IKKβ cKO mice infected with 10^6 ^CFU of *Ft*. LVS i.d. and analyzed by flow cytometry for M1 activation. (**A**) Pro-inflammatory macrophages were identified by their F4/80 and macrosialin (CD68) expression and then scored for M1 activation by (**B**) IL-12 expression. Statistical analysis was performed by one-way ANOVA followed by Tukey’s ad hoc post-test. (n for IKK*^f/f^*, IKKα cKO, IKKβ cKO on day 0∶5/5/5; day 2∶4/4/4; day 6∶4/4/4; day 8∶4/4/3). Results are representative of at least two independent experiments. Bars represent the mean ± SD. **P<0.01.

**Figure 9:**
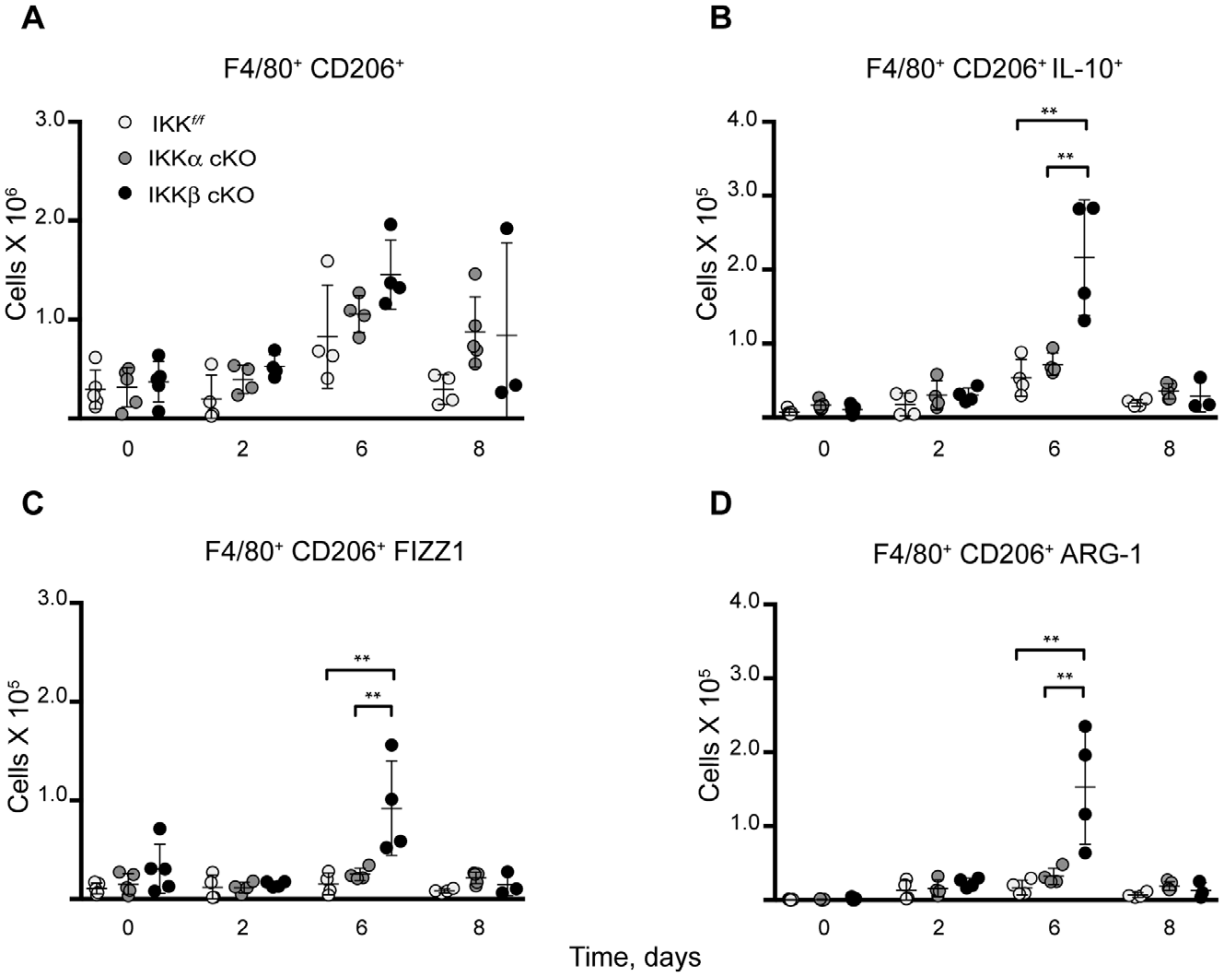
Switch to M2a polarization in IKKβ cKO mice at mid-infection stage. M2a activation occurs in IKKβ cKO mice after sublethal i.d. infection with 10^6^ *Ft*. LVS as evidenced by expression of (**A**) CD206 (**B**) IL-10 (**C**) FIZZ1/Relmα and (**D**) Arg-1 in a flow cytometry time course experiment. Statistical analysis was performed by one-way ANOVA followed by Tukey’s ad hoc post-test. (n for IKK*^f/f^*, IKKα cKO, IKKβ cKO on day 0∶5/5/5; day 2∶4/4/4; day 6∶4/4/4; day 8∶4/4/3). Results are representative of at least two independent experiments. Bars represent the mean ± SD. **P<0.01.

IKKβ deficiency in myeloid cells was previously reported to induce classical/M1 macrophage polarization in response to GBS infection [11], we, therefore, questioned whether this also occurs in the liver in response to *Ft*. LVS infection. To address this, mice were injected i.d. with 10^6^ CFU of *Ft*. LVS and we analyzed the NPC fraction isolated from the livers of infected mice in a time course experiment using flow cytometry. To first broadly identify M1 and M2a macrophage subpopulations, we scored macrophages for their F4/80 expression and either macrosialin (murine CD68) or the mannose receptor (CD206), (Fig. 8A and 9A, respectively). Next, we evaluated each subpopulation for expression of the prototypical M1 cytokine, IL-12 (Fig. 8B) or multiple M2 activation markers, including IL-10, arginase-1 and RELMα/FIZZ1 (Fig. 9B–D).

We observed a 5-fold increase (P = 0.0040) in the number of M1 macrophages (F4/80^+^ CD68^+^ IL-12^+^) in IKKβ cKO mice at 2 days post-infection (Fig. 8B). This data indicates that myeloid IKKβ contributes to the suppression of M1 classical activation in the liver in response to sublethal *Ft*. LVS infection. However, this IKKβ-mediated M1 suppression only occurred in a short time period early in infection, as by day 6 there was a shift toward M2a activation as indicated by increases in F4/80^+^ CD206^+^ staining in coordination with several other M2a-associated markers (Fig. 9B–D) including: IL-10, a 2 fold increase P = 0.0019; FIZZ1, a 6 fold increase P = 0.0085; and Arg-1, a 9 fold increase P = 0.0041).

### Livers of *Ft.* LVS Infected IKKβ cKO Mice Present Protracted Activation of CD8^+^ T cells in the Liver

**Figure 10:**
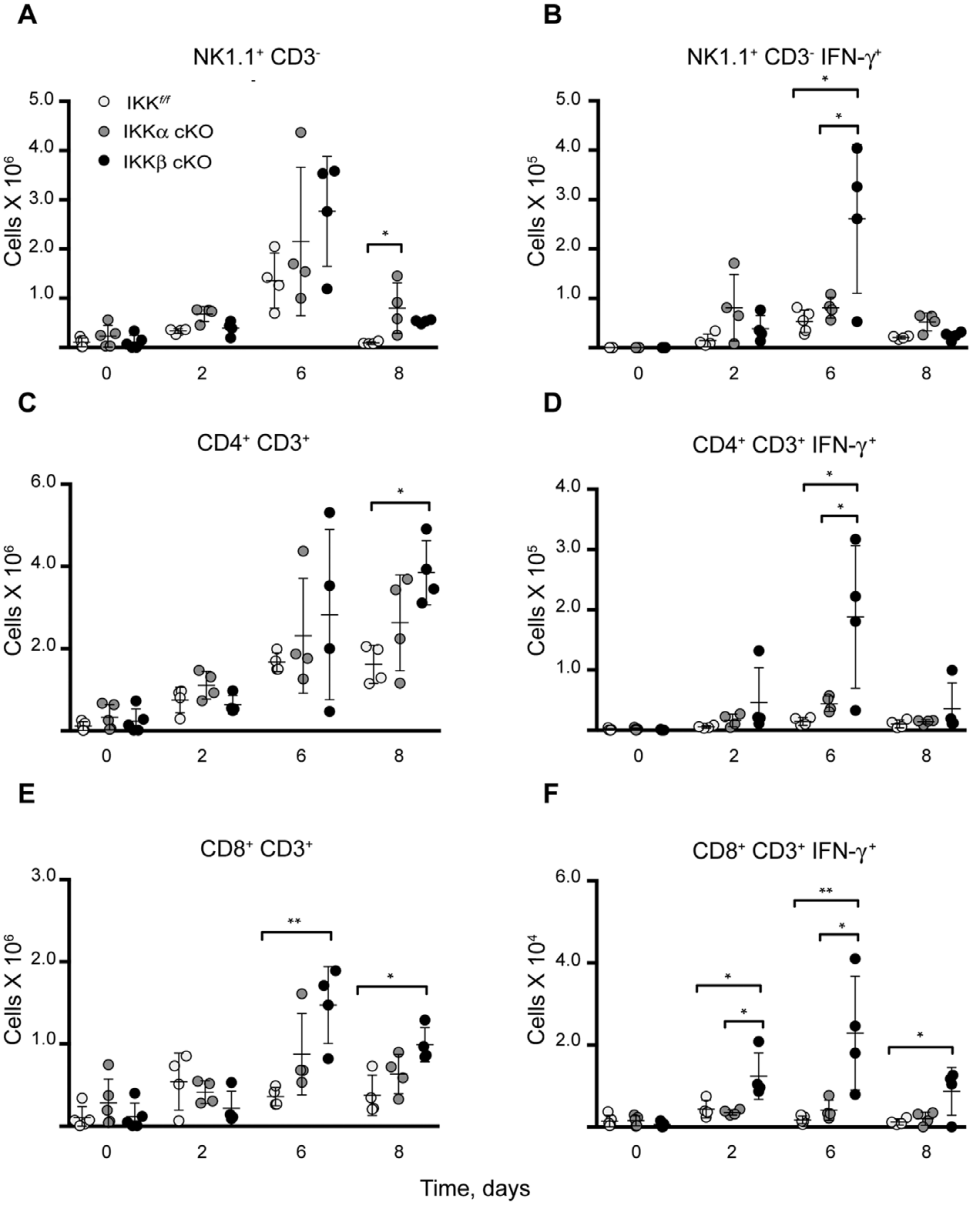
Protracted hepatic activation of CD8^+^ T cells in IKKβ cKO *Ft.* LVS infected mice. Hepatic (**A**) Natural killer (NK1.1^+^CD3^−^), (**B**) CD4^+^CD3^+^ and (**C**) CD8^+^CD3^+^ T-lymphocytes and their (**D–F**) IFN-γ expression, respectively, were analyzed by flow cytometry in *Ft.* LVS infected mice. Statistical analysis was performed by one-way ANOVA followed by Tukey’s ad hoc post-test. (n for IKK*^f/f^*, IKKα cKO, IKKβ cKO on day 0∶5/5/5; day 2∶4/4/4; day 6∶4/4/4; day 8∶4/4/3). All results are representative of at least two independent experiments. Bars represent the mean ± SD. *P<0.05; **P<0.01.

We next evaluated the collateral effects of IKK myeloid deletion with respect to the activation of IFN-γ by other cell types after sublethal i.d. *Ft*. LVS infection. Again, using a flow cytometric approach on liver NPCs, we scored for IFN-γ activation in NK (NK1.1^+^ CD3^−^), CD4^+^ and CD8^+^ T cell (CD4^+^ CD3^+^ and CD8^+^ CD3^+^, respectively) populations. The NK cell populations from IKKα and IKKβ cKO infected mice were largely similar to IKK*^f/f^* control animals (Fig. 10A). A small increase in NK cells were seen in IKKα cKO animals on day 8 post-infection, yet we did not observe corresponding increases in IFN-γ^+^ cells at the same time point (Fig. 10B). Increases in both CD4^+^ and CD8^+^ T cell populations were observed at later time points in infection in IKKβ cKO mice (Fig. 10C and E). In CD8^+^ T cells, these increases correspondingly correlated to greater increases in the number of IFN-γ^+^ cells (Fig. 10E and F). Interestingly, increases in IFN-γ^+^ CD8^+^ T cells from IKKβ cKO infected mice were protracted throughout the entire infection time course (Fig. 10F). This trend was not observed in IKK*^f/f^* or IKKα cKO infected mice. These results suggest that myeloid IKKβ may function in an extrinsic manner to suppress the influx of activated IFN-γ producing cells.

## Discussion

The results of this work shed new light on the contributions of the IKKs in myeloid cells during *Ft*. LVS infection and also extend previous findings on the *in vivo* roles of the IKKs in myeloid cells in response to bacterial infections. In spite of the anti-inflammatory properties demonstrated by others [9], [10], [11], myeloid IKKs exhibit notable differences in response to the intracellular bacterium *Ft*. LVS. Here, we found that myeloid IKKβ has a protective role in response to lethal challenge. At sub-lethal doses, histopathology and retroplate culture assays showed that myeloid IKKβ helps regulate the granulomatous response and control infection.

We also examined the effect loss myeloid IKKβ had on macrophage polarization. Indeed, IKKβ inhibits classical M1 macrophage activation. However, this effect was limited to early time points of infection, in spite of a protracted elevation of IFN-γ^+^ CD8^+^ T cells during the course of infection. On the contrary, we observed only limited roles for myeloid IKKα during *Ft*. LVS infection. Loss of myeloid IKKα resulted in similar survival rates compared to control mice and a reduction in bacterial colonization at early time points. The most notable difference in IKKα cKO mice was in the appearance of large, necrotic, granuloma-like foci, which formed in the liver at late stages of infection, suggesting that IKKα in myeloid cells may be involved in clearance of dying cells. These foci were cleared after 90 days of infection (data not shown) by a yet unknown mechanism.

### IKKβ but not IKKα in Myeloid Cells Contributes to Host Survival to a Lethal Dose of *Ft.* LVS

The survival results we obtained in our study are in contrast to those reported for myeloid IKK deficient mice responding to a lethal Gram-positive bacterium GBS (Group B *Streptococcus*) challenge [10], [11]. In this previous study, mice devoid of myeloid IKKβ were more resistant to a lethal challenge with GBS [11]. Here, we found that intradermal inoculation with Gram-negative bacterium, *Ft.* LVS rendered myeloid IKKβ deficient mice much more susceptible to lethal infection. These results correspondingly correlated to levels of bacterial titers, as there was increased bacterial clearance in the GBS model [11], while we observed increased colonization in the *Ft*. LVS model. In addition, loss of functional IKKα (*ikkα* ^AA/AA^ phosphorylation mutant) in the myeloid compartment results in increased susceptibility to GBS infection [10], while here, we found IKKα cKO mice maintain a comparable susceptibility in response to *Ft*. LVS infection to control animals. These contrasting results in survival between the *Francisella* and GBS infection models in myeloid IKK deficient mice suggest that there are differential requirements for downstream TLR effectors, such as the IKKs, during septic challenge. In fact, similar differences in survival patterns have been reported for the TLR adapter protein MyD88 (Myeloid-differentiation factor 88). Mice deficient in MyD88 are protected from GBS infection at high infection doses, [61], while *Ft*. LVS infected mice are extremely susceptible to MyD88 loss at low doses [55]. Moreover, increased mortality occurred in myeloid IKKβ deficient mice at the LD_50_ dose of 10^8^ CFU of *Ft*. LVS, indicating a protective role for myeloid IKKβ during overwhelming sepsis. IKKβ deficiency also resulted in a dose-dependent survival response, an effect which has been observed, to varying degrees, for other components involved in NF-κB signaling. For example, loss of either TLR2 (Toll-like receptor 2) [62] or MyD88 [55] results in reduced survival as a result of tularemic infection. Yet, loss of TLR2 affects survival at moderate infection doses (∼4×10^4^ CFU) [62], while MyD88 deficient mice are extremely sensitive and succumb to *Ft*. LVS at infection doses orders of magnitude lower (5×10^1^ CFU) [55]. The increased sensitivity of MyD88 loss is due to its requirement in multiple signaling pathways. Studies show that MyD88 loss affects the NF-κB, c-Jun and p38 MAPK pathways with delayed kinetics [63], abrogated IL-1R- and IL-18R -mediated cytokine production [64], as well as impaired IFN-γ and TNF-α production in response to several pathogens [63], [64]. In addition, it is important to note that the dose-specific sensitivity to *Ft*. LVS in the TLR2 and MyD88 studies were performed in full knockout models, while the IKKβ model used here was conditionally restricted to the myeloid compartment. Thus, this is the first study to show a myeloid-specific requirement for host survival in response to *Francisella tularensis.*

### *Ft.* LVS Disseminates to Lung, Liver and Spleen

Dissemination of bacteria led to bacterial colonization in lung, spleen and liver tissues. Only mild histopathological findings in lung tissue were found. One unexpected finding was the development of organizing lymphocytic aggregates within the lungs IKKβ cKO mice after infection. These aggregates, also known as iBALT (inducible bronchus-associated lymphoid tissue), are not present in normal lung in humans or mice, but can be induced after exposure to antigen, inflammation or infection (reviewed in [65], [66]). In mice lacking spleen, lymph nodes and Peyer’s patches, iBALT acts as a site of B and T cell proliferation in response to influenza infection and is considered protective in nature [54]. iBALT develops under pathologic states, and it is suggested that this may involve the activation of PRR (pattern recognition receptors) such as the TLRs [65]. Although further examination into the development of iBALT structures in IKKβ cKO mouse lung was beyond the scope of this current investigation, it is of note that these structures were previously identified in a number of *Ft*. LVS vaccine studies. Organized lymphoid structures are induced upon aerosol or intranasal administration of vaccines using whole [67], inactivated *Ft*. LVS [68] or *Ft*. LVS LPS plus adjuvant [69]. However, unlike these studies, where mice were immunized with several repeated doses of bacteria, our model consisted of a single intradermal inoculation. It is possible that the increased bacterial burden in IKKβ cKO mice, induces these structures as a result of ongoing and repeated exposure to *Ft*. antigen. Furthermore, iBALT structures persist in these mice well after bacterial clearance and are still evident at 90 days post infection (S.S., unpublished data).

IKKβ cKO mice responded to infection with an increased frequency in granuloma response in both spleen and liver. IKKα cKO mice developed some abnormally large granulomas within liver; however, the spleen granuloma reaction was qualitatively and quantitatively similar to IKK*^f/f^* mice. Analysis of *Ft*. antigen in the liver showed the bacteria were mainly localized within granulomas, although a limited number of infected cells could be detected within the parenchyma.

In the liver, iNOS (inducible nitric oxide synthase) is produced by macrophages and hepatocytes and can be induced by microbial lipoproteins through TLR activation, IFN-γ or TNF-α. iNOS mediates the metabolic conversion of L-arginine to NO (nitric oxide) and citrulline. NO is not only a powerful antimicrobial effector molecule capable of direct killing, it is also involved in several other aspects of host defense including cytokine production and apoptosis [70]. iNOS expression within granulomas was previously reported to be low and infrequent during intradermal infection [24]. However, we found 40–54% of hepatic granulomas were positive for iNOS induction by day 2, with only marginal changes in expression throughout the course of infection. Thus, it is unlikely that the observed defects in granuloma maintenance result from aberrant iNOS induction.

Apoptosis is considered an important immune defense mechanism. Rapid elimination of infected cells helps prevent the spread of infection to other cells and also promotes dendritic cell uptake of apoptotic bodies, allowing access to antigen which may trigger both innate and adaptive immune responses. However, many pathogens have evolved mechanisms to escape destruction by subverting or redirecting these processes (reviewed in [71]). We found the early apoptosis marker, cleaved caspase-3 (CC-3), spatially localized within the granulomas in all three strains of mice. We found no overt differences between strains in the percentage of CC-3-positve granulomas at early time points. Furthermore, only a few cells per granuloma stained positive for this marker, but this was consistent with other models of intradermal *Ft*. LVS infection [51]. Lawrence *et al* suggests that increased bactericidal control in a GBS model of pneumonia observed in *ikkα* ^AA/AA^ macrophages is due, in part, to increased activation of anti-apoptotic genes combined with sustained activation of inflammatory genes such as iNOS [10]. In the *Ft*. LVS model, these effects on apoptosis and iNOS production were not evident, suggesting that the molecular mechanisms between these two infection models are different. Furthermore, GBS-infected *ikkα* ^AA/AA^ mice, despite increased bacterial clearance at early time points, eventually succumb to infection, whereas survival in IKKα cKO mice infected at the LD_50_ dose of 10^8^ CFU of *Ft*. LVS are similar to wild type mice.

### IKKβ Deficiency in Myeloid Cells Causes Time Dependent, Differential Effects on M1 *vs* M2 Macrophage Polarization in Response to Sublethal *Ft*. LVS Infection

Despite differences in survival, the observation that myeloid IKKβ deficiency induces M1/classical activation in the livers of *Ft*. LVS infected mice is in accord with the GBS infection model [11]. Because our model system allows us to monitor macrophage polarization over days, not hours (as is the case in GBS model), we also discovered that IKKβ cKO mice undergo a second wave of polarization toward M2/alternatively activated macrophages at the mid-infection stage. Although we cannot rule out that this second wave of polarization stems from secondary or bystander effects induced by the local environment, it is of note that the anti-inflammatory IKKβ-dependent blockade of M1 classical macrophage activation is transient in nature and thus an important consideration for design of therapeutic targeting.

### IKKβ Loss in Myeloid Cells Results in Collateral Effects on IFN-γ Producing Cells

The compounding effects of increased bacterial titers and M1/M2 activation defects in IKKβ cKO mice also correlated with increases in several types of IFN-γ expressing cells. Furthermore, protracted elevations in IFN-γ expressing CD8^+^ T cells were protracted throughout the course of infection. Elevations of these cells persisted, despite the mid-infection shift in M2 macrophage polarization. It has long been considered that early tularemic infection is controlled by T cell-independent events, as T cell depleted mice (depleted of both CD4^+^ and CD8^+^ T cells) survive initial infection, but fail to clear bacteria and succumb to infection several weeks after inoculation [72], [73], [74]. Interestingly, mice selectively depleted for only CD4^+^ T cells or CD8^+^ T cells are able to resolve and survive infection [75]. This implies that these T cell subsets have roles in clearance and resolution of infection that largely overlap. Although the exact mechanism(s) by which myeloid IKKβ deficiency results in a collateral elevation of reactive CD8^+^ T cells remains to be determined, our data suggest that when myeloid NF-κB is impaired due to loss of IKKβ, CD8^+^ T cells overtly respond as an immunologic compensatory mechanism. Therefore, it seems that CD8^+^ T cells are poised to react during the T-cell independent phase of *Ft*. infection.

In summary, we show that myeloid IKKβ has a protective role in controlling bacterial growth that leads to overwhelming sepsis during tularemic infection. Furthermore, macrophage polarization *vs*. bacterial control and survival appear to be functionally distinct processes regulated by myeloid IKKβ. In addition, we also found, in our model system, that IKKβ-dependent macrophage polarization is a short-term phenomenon. Contrary to this, we found no evidence of anti-inflammatory properties for myeloid IKKα *in vivo*.

## Supporting Information

Figure S1 **LD_50_ and sublethal** ***Ft*** **. LVS dose determinations. (A)** IKK*^f/f^* control mice (n = 5 mice/group) were injected with *Ft.* LVS i.d. at the indicated doses and analyzed for survival by Kaplan-Meier method in order to determine the median lethal dose. The inoculation dose was confirmed by retroplate assay and the LD_50_ was estimated at 10^8^ CFU. In panels (**B)** and **(C)**, IKK*^f/f^*, IKKα cKO and IKKβ cKO mice were injected with *Ft*. LVS i.d at two different sublethal doses: **(B)** 10^7^ CFU and **(C)** 10^6^ CFU, and analyzed for long-term survival (n = 5–6 mice/group).
(TIF)

Figure S2 **Minimal lung involvement after intradermal** ***Ft*** **. LVS challenge.** Lung sections, taken from mice i.d. challenged with a sublethal dose of 10^6^ CFU *Ft*. LVS, were analyzed by immunohistochemistry for *Ft*. LVS antigen. **(A)** and **(D)** IKK*^f/f^*, **(B)** and **(E)** IKKα cKO, and **(C)** and **(F)** IKKβ cKO are representative lung sections showing low *Ft*. LVS colonization at days 2 and 9 post infection, respectively. Magnification = 100×, scale bar = 100 µm.
(TIF)

Figure S3 ***Ft*** **. LVS dissemination in spleen.** Spleen sections from mice i.d. challenged with a sublethal dose of 10^6^ CFU *Ft*. LVS, were analyzed by immunohistochemistry for *Ft*. LVS antigen. **(A)** and **(D)** IKK*^f/f^*, **(B)** and **(E)** IKKα cKO, and **(C)** and **(F)** IKKβ cKO are representative sections of spleens at days 2 and 9 post infection, respectively. Magnification = 100×, scale bar = 100 µm.
(TIF)

## References

[1] Israel A (2010) The IKK complex, a central regulator of NF-kappaB activation. Cold Spring Harb Perspect Biol 2: a000158. 20300203 10.1101/cshperspect.a000158 PMC2829958

[2] Scheidereit C (2006) IkappaB kinase complexes: gateways to NF-kappaB activation and transcription. Oncogene 25: 6685–6705. 17072322 10.1038/sj.onc.1209934

[3] Ghosh S, Hayden MS (2008) New regulators of NF-kappaB in inflammation. Nat Rev Immunol 8: 837–848. 18927578 10.1038/nri2423

[4] Vallabhapurapu S, Karin M (2009) Regulation and function of NF-kappaB transcription factors in the immune system. Annu Rev Immunol 27: 693–733. 19302050 10.1146/annurev.immunol.021908.132641

[5] Perkins ND (2007) Integrating cell-signalling pathways with NF-kappaB and IKK function. Nat Rev Mol Cell Biol 8: 49–62. 17183360 10.1038/nrm2083

[6] Chariot A (2009) The NF-kappaB-independent functions of IKK subunits in immunity and cancer. Trends Cell Biol 19: 404–413. 19648011 10.1016/j.tcb.2009.05.006

[7] Timmer AM, Nizet V (2008) IKKbeta/NF-kappaB and the miscreant macrophage. J Exp Med 205: 1255–1259. 18519650 10.1084/jem.20081056 PMC2413023

[8] Liu F, Xia Y, Parker AS, Verma IM (2012) IKK biology. Immunol Rev 246: 239–253. 22435559 10.1111/j.1600-065X.2012.01107.x PMC3311052

[9] Li Q, Lu Q, Bottero V, Estepa G, Morrison L, et al (2005) Enhanced NF-kappaB activation and cellular function in macrophages lacking IkappaB kinase 1 (IKK1). Proc Natl Acad Sci U S A 102: 12425–12430. 16116086 10.1073/pnas.0505997102 PMC1194954

[10] Lawrence T, Bebien M, Liu GY, Nizet V, Karin M (2005) IKKalpha limits macrophage NF-kappaB activation and contributes to the resolution of inflammation. Nature 434: 1138–1143. 15858576 10.1038/nature03491

[11] Fong CH, Bebien M, Didierlaurent A, Nebauer R, Hussell T, et al (2008) An antiinflammatory role for IKKbeta through the inhibition of “classical” macrophage activation. J Exp Med 205: 1269–1276. 18490491 10.1084/jem.20080124 PMC2413025

[12] Lawrence T, Fong C (2010) The resolution of inflammation: anti-inflammatory roles for NF-kappaB. Int J Biochem Cell Biol 42: 519–523. 20026420 10.1016/j.biocel.2009.12.016

[13] Fortier AH, Slayter MV, Ziemba R, Meltzer MS, Nacy CA (1991) Live vaccine strain of Francisella tularensis: infection and immunity in mice. Infect Immun 59: 2922–2928. 1879918 10.1128/iai.59.9.2922-2928.1991 PMC258114

[14] Rick Lyons C, Wu TH (2007) Animal models of Francisella tularensis infection. Ann N Y Acad Sci 1105: 238–265. 17395735 10.1196/annals.1409.003

[15] Bosio CM, Dow SW (2005) Francisella tularensis induces aberrant activation of pulmonary dendritic cells. J Immunol 175: 6792–6801. 16272336 10.4049/jimmunol.175.10.6792

[16] Gavrilin MA, Bouakl IJ, Knatz NL, Duncan MD, Hall MW, et al (2006) Internalization and phagosome escape required for Francisella to induce human monocyte IL-1beta processing and release. Proc Natl Acad Sci U S A 103: 141–146. 16373510 10.1073/pnas.0504271103 PMC1324976

[17] Sjostedt A, Conlan JW, North RJ (1994) Neutrophils are critical for host defense against primary infection with the facultative intracellular bacterium Francisella tularensis in mice and participate in defense against reinfection. Infect Immun 62: 2779–2783. 8005668 10.1128/iai.62.7.2779-2783.1994 PMC302881

[18] McCaffrey RL, Allen LA (2006) Francisella tularensis LVS evades killing by human neutrophils via inhibition of the respiratory burst and phagosome escape. J Leukoc Biol 80: 1224–1230. 16908516 10.1189/jlb.0406287 PMC1828114

[19] Conlan JW, North RJ (1992) Early pathogenesis of infection in the liver with the facultative intracellular bacteria Listeria monocytogenes, Francisella tularensis, and Salmonella typhimurium involves lysis of infected hepatocytes by leukocytes. Infect Immun 60: 5164–5171. 1452350 10.1128/iai.60.12.5164-5171.1992 PMC258293

[20] Hall JD, Craven RR, Fuller JR, Pickles RJ, Kawula TH (2006) Francisella tularensis Replicates Within Alveolar Type II Epithelial Cells in vitro and in vivo Following Inhalation. Infect Immun: IAI.01254-01206. 10.1128/IAI.01254-06 PMC1828526 17088343

[21] Fortier AH, Polsinelli T, Green SJ, Nacy CA (1992) Activation of macrophages for destruction of Francisella tularensis: identification of cytokines, effector cells, and effector molecules. Infect Immun 60: 817–825. 1541555 10.1128/iai.60.3.817-825.1992 PMC257560

[22] Hall JD, Woolard MD, Gunn BM, Craven RR, Taft-Benz S, et al (2008) Infected-host-cell repertoire and cellular response in the lung following inhalation of Francisella tularensis Schu S4, LVS, or U112. Infect Immun 76: 5843–5852. 18852251 10.1128/IAI.01176-08 PMC2583552

[23] Conlan JW, Chen W, Shen H, Webb A, KuoLee R (2003) Experimental tularemia in mice challenged by aerosol or intradermally with virulent strains of Francisella tularensis: bacteriologic and histopathologic studies. Microb Pathog 34: 239–248. 12732472 10.1016/s0882-4010(03)00046-9

[24] Cole LE, Elkins KL, Michalek SM, Qureshi N, Eaton LJ, et al (2006) Immunologic consequences of Francisella tularensis live vaccine strain infection: role of the innate immune response in infection and immunity. J Immunol 176: 6888–6899. 16709849 10.4049/jimmunol.176.11.6888

[25] Bosio CM, Bielefeldt-Ohmann H, Belisle JT (2007) Active suppression of the pulmonary immune response by Francisella tularensis Schu4. J Immunol 178: 4538–4547. 17372012 10.4049/jimmunol.178.7.4538

[26] Conlan JW, Zhao X, Harris G, Shen H, Bolanowski M, et al (2008) Molecular immunology of experimental primary tularemia in mice infected by respiratory or intradermal routes with type A Francisella tularensis. Mol Immunol 45: 2962–2969. 18321578 10.1016/j.molimm.2008.01.022 PMC2715917

[27] Sandstrom G, Lofgren S, Tarnvik A (1988) A capsule-deficient mutant of Francisella tularensis LVS exhibits enhanced sensitivity to killing by serum but diminished sensitivity to killing by polymorphonuclear leukocytes. Infect Immun 56: 1194–1202. 3356465 10.1128/iai.56.5.1194-1202.1988 PMC259783

[28] Ben Nasr A, Klimpel GR (2008) Subversion of complement activation at the bacterial surface promotes serum resistance and opsonophagocytosis of Francisella tularensis. J Leukoc Biol 84: 77–85. 18430786 10.1189/jlb.0807526

[29] Clay CD, Soni S, Gunn JS, Schlesinger LS (2008) Evasion of complement-mediated lysis and complement C3 deposition are regulated by Francisella tularensis lipopolysaccharide O antigen. J Immunol 181: 5568–5578. 18832715 10.4049/jimmunol.181.8.5568 PMC2782685

[30] Anthony LD, Burke RD, Nano FE (1991) Growth of Francisella spp. in rodent macrophages. Infect Immun 59: 3291–3296. 1879943 10.1128/iai.59.9.3291-3296.1991 PMC258167

[31] Santic M, Molmeret M, Klose KE, Jones S, Kwaik YA (2005) The Francisella tularensis pathogenicity island protein IglC and its regulator MglA are essential for modulating phagosome biogenesis and subsequent bacterial escape into the cytoplasm. Cell Microbiol 7: 969–979. 15953029 10.1111/j.1462-5822.2005.00526.x

[32] Clemens DL, Lee BY, Horwitz MA (2005) Francisella tularensis enters macrophages via a novel process involving pseudopod loops. Infect Immun 73: 5892–5902. 16113308 10.1128/IAI.73.9.5892-5902.2005 PMC1231130

[33] Santic M, Molmeret M, Klose KE, Abu Kwaik Y (2006) Francisella tularensis travels a novel, twisted road within macrophages. Trends Microbiol 14: 37–44. 16356719 10.1016/j.tim.2005.11.008

[34] Vinogradov E, Perry MB, Conlan JW (2002) Structural analysis of Francisella tularensis lipopolysaccharide. Eur J Biochem 269: 6112–6118. 12473106 10.1046/j.1432-1033.2002.03321.x

[35] Chen W, Kuolee R, Shen H, Busa M, Conlan JW (2005) Toll-like receptor 4 (TLR4) plays a relatively minor role in murine defense against primary intradermal infection with Francisella tularensis LVS. Immunol Lett 97: 151–154. 15626487 10.1016/j.imlet.2004.10.001

[36] Duenas AI, Aceves M, Orduna A, Diaz R, Sanchez Crespo M, et al (2006) Francisella tularensis LPS induces the production of cytokines in human monocytes and signals via Toll-like receptor 4 with much lower potency than E. coli LPS. Int Immunol 18: 785–795. 16574669 10.1093/intimm/dxl015

[37] Katz J, Zhang P, Martin M, Vogel SN, Michalek SM (2006) Toll-like receptor 2 is required for inflammatory responses to Francisella tularensis LVS. Infect Immun 74: 2809–2816. 16622218 10.1128/IAI.74.5.2809-2816.2006 PMC1459727

[38] Li H, Nookala S, Bina XR, Bina JE, Re F (2006) Innate immune response to Francisella tularensis is mediated by TLR2 and caspase-1 activation. J Leukoc Biol 80: 766–773. 16895974 10.1189/jlb.0406294

[39] Cole LE, Shirey KA, Barry E, Santiago A, Rallabhandi P, et al (2007) Toll-like receptor 2-mediated signaling requirements for Francisella tularensis live vaccine strain infection of murine macrophages. Infect Immun 75: 4127–4137. 17517865 10.1128/IAI.01868-06 PMC1951974

[40] Hong KJ, Wickstrum JR, Yeh HW, Parmely MJ (2007) Toll-like receptor 2 controls the gamma interferon response to Francisella tularensis by mouse liver lymphocytes. Infect Immun 75: 5338–5345. 17785474 10.1128/IAI.00561-07 PMC2168295

[41] Telepnev M, Golovliov I, Grundstrom T, Tarnvik A, Sjostedt A (2003) Francisella tularensis inhibits Toll-like receptor-mediated activation of intracellular signalling and secretion of TNF-alpha and IL-1 from murine macrophages. Cell Microbiol 5: 41–51. 12542469 10.1046/j.1462-5822.2003.00251.x

[42] Telepnev M, Golovliov I, Sjostedt A (2005) Francisella tularensis LVS initially activates but subsequently down-regulates intracellular signaling and cytokine secretion in mouse monocytic and human peripheral blood mononuclear cells. Microb Pathog 38: 239–247. 15925273 10.1016/j.micpath.2005.02.003

[43] Shirey KA, Cole LE, Keegan AD, Vogel SN (2008) Francisella tularensis live vaccine strain induces macrophage alternative activation as a survival mechanism. J Immunol 181: 4159–4167. 18768873 10.4049/jimmunol.181.6.4159 PMC2637804

[44] Gordon S (2003) Alternative activation of macrophages. Nat Rev Immunol 3: 23–35. 12511873 10.1038/nri978

[45] Martinez FO, Sica A, Mantovani A, Locati M (2008) Macrophage activation and polarization. Front Biosci 13: 453–461. 17981560 10.2741/2692

[46] Parsa KV, Butchar JP, Rajaram MV, Cremer TJ, Gunn JS, et al (2008) Francisella gains a survival advantage within mononuclear phagocytes by suppressing the host IFNgamma response. Mol Immunol 45: 3428–3437. 18514317 10.1016/j.molimm.2008.04.006 PMC2577832

[47] Penzo M, Molteni R, Suda T, Samaniego S, Raucci A, et al (2010) Inhibitor of NF-kappa B kinases alpha and beta are both essential for high mobility group box 1-mediated chemotaxis [corrected]. J Immunol 184: 4497–4509. 20231695 10.4049/jimmunol.0903131 PMC2915896

[48] Golovliov I, Sandstrom G, Ericsson M, Sjostedt A, Tarnvik A (1995) Cytokine expression in the liver during the early phase of murine tularemia. Infect Immun 63: 534–538. 7822019 10.1128/iai.63.2.534-538.1995 PMC173028

[49] Clausen BE, Burkhardt C, Reith W, Renkawitz R, Forster I (1999) Conditional gene targeting in macrophages and granulocytes using LysMcre mice. Transgenic Res 8: 265–277. 10621974 10.1023/a:1008942828960

[50] Forestal CA, Benach JL, Carbonara C, Italo JK, Lisinski TJ, et al (2003) Francisella tularensis selectively induces proinflammatory changes in endothelial cells. J Immunol 171: 2563–2570. 12928407 10.4049/jimmunol.171.5.2563

[51] Rasmussen JW, Cello J, Gil H, Forestal CA, Furie MB, et al (2006) Mac-1+ cells are the predominant subset in the early hepatic lesions of mice infected with Francisella tularensis. Infect Immun 74: 6590–6598. 17000726 10.1128/IAI.00868-06 PMC1698106

[52] Egen JG, Rothfuchs AG, Feng CG, Winter N, Sher A, et al (2008) Macrophage and T cell dynamics during the development and disintegration of mycobacterial granulomas. Immunity 28: 271–284. 18261937 10.1016/j.immuni.2007.12.010 PMC2390753

[53] Chen W, Shen H, Webb A, KuoLee R, Conlan JW (2003) Tularemia in BALB/c and C57BL/6 mice vaccinated with Francisella tularensis LVS and challenged intradermally, or by aerosol with virulent isolates of the pathogen: protection varies depending on pathogen virulence, route of exposure, and host genetic background. Vaccine 21: 3690–3700. 12922099 10.1016/s0264-410x(03)00386-4

[54] Moyron-Quiroz JE, Rangel-Moreno J, Kusser K, Hartson L, Sprague F, et al (2004) Role of inducible bronchus associated lymphoid tissue (iBALT) in respiratory immunity. Nat Med 10: 927–934. 15311275 10.1038/nm1091

[55] Collazo CM, Sher A, Meierovics AI, Elkins KL (2006) Myeloid differentiation factor-88 (MyD88) is essential for control of primary in vivo Francisella tularensis LVS infection, but not for control of intra-macrophage bacterial replication. Microbes Infect 8: 779–790. 16513388 10.1016/j.micinf.2005.09.014

[56] Bokhari SM, Kim KJ, Pinson DM, Slusser J, Yeh HW, et al (2008) NK cells and gamma interferon coordinate the formation and function of hepatic granulomas in mice infected with the Francisella tularensis live vaccine strain. Infect Immun 76: 1379–1389. 18227174 10.1128/IAI.00745-07 PMC2292861

[57] Fortier AH, Green SJ, Polsinelli T, Jones TR, Crawford RM, et al (1994) Life and death of an intracellular pathogen: Francisella tularensis and the macrophage. Immunol Ser 60: 349–361. 8251580

[58] Lindgren H, Stenmark S, Chen W, Tarnvik A, Sjostedt A (2004) Distinct roles of reactive nitrogen and oxygen species to control infection with the facultative intracellular bacterium Francisella tularensis. Infect Immun 72: 7172–7182. 15557642 10.1128/IAI.72.12.7172-7182.2004 PMC529105

[59] Brightbill HD, Libraty DH, Krutzik SR, Yang RB, Belisle JT, et al (1999) Host defense mechanisms triggered by microbial lipoproteins through toll-like receptors. Science 285: 732–736. 10426995 10.1126/science.285.5428.732

[60] Ramirez-Carrozzi VR, Braas D, Bhatt DM, Cheng CS, Hong C, et al (2009) A unifying model for the selective regulation of inducible transcription by CpG islands and nucleosome remodeling. Cell 138: 114–128. 19596239 10.1016/j.cell.2009.04.020 PMC2712736

[61] Mancuso G, Midiri A, Beninati C, Biondo C, Galbo R, et al (2004) Dual role of TLR2 and myeloid differentiation factor 88 in a mouse model of invasive group B streptococcal disease. J Immunol 172: 6324–6329. 15128822 10.4049/jimmunol.172.10.6324

[62] Abplanalp AL, Morris IR, Parida BK, Teale JM, Berton MT (2009) TLR-dependent control of Francisella tularensis infection and host inflammatory responses. PLoS One 4: e7920. 19936231 10.1371/journal.pone.0007920 PMC2775407

[63] Kawai T, Adachi O, Ogawa T, Takeda K, Akira S (1999) Unresponsiveness of MyD88-deficient mice to endotoxin. Immunity 11: 115–122. 10435584 10.1016/s1074-7613(00)80086-2

[64] Adachi O, Kawai T, Takeda K, Matsumoto M, Tsutsui H, et al (1998) Targeted disruption of the MyD88 gene results in loss of IL-1- and IL-18-mediated function. Immunity 9: 143–150. 9697844 10.1016/s1074-7613(00)80596-8

[65] Foo SY, Phipps S (2010) Regulation of inducible BALT formation and contribution to immunity and pathology. Mucosal Immunol 3: 537–544. 20811344 10.1038/mi.2010.52

[66] Neyt K, Perros F, GeurtsvanKessel CH, Hammad H, Lambrecht BN (2012) Tertiary lymphoid organs in infection and autoimmunity. Trends Immunol 33: 297–305. 22622061 10.1016/j.it.2012.04.006 PMC7106385

[67] Wayne Conlan J, Shen H, Kuolee R, Zhao X, Chen W (2005) Aerosol-, but not intradermal-immunization with the live vaccine strain of Francisella tularensis protects mice against subsequent aerosol challenge with a highly virulent type A strain of the pathogen by an alphabeta T cell- and interferon gamma- dependent mechanism. Vaccine 23: 2477–2485. 15752834 10.1016/j.vaccine.2004.10.034

[68] Baron SD, Singh R, Metzger DW (2007) Inactivated Francisella tularensis live vaccine strain protects against respiratory tularemia by intranasal vaccination in an immunoglobulin A-dependent fashion. Infect Immun 75: 2152–2162. 17296747 10.1128/IAI.01606-06 PMC1865787

[69] Chiavolini D, Rangel-Moreno J, Berg G, Christian K, Oliveira-Nascimento L, et al (2010) Bronchus-associated lymphoid tissue (BALT) and survival in a vaccine mouse model of tularemia. PLoS One 5: e11156. 20585390 10.1371/journal.pone.0011156 PMC2886834

[70] Bogdan C, Röllinghoff M, Diefenbach A (2000) Reactive oxygen and reactive nitrogen intermediates in innate and specific immunity. Current Opinion in Immunology 12: 64–76. 10679404 10.1016/s0952-7915(99)00052-7

[71] Ashida H, Mimuro H, Ogawa M, Kobayashi T, Sanada T, et al (2011) Cell death and infection: a double-edged sword for host and pathogen survival. J Cell Biol 195: 931–942. 22123830 10.1083/jcb.201108081 PMC3241725

[72] Leiby DA, Fortier AH, Crawford RM, Schreiber RD, Nacy CA (1992) In vivo modulation of the murine immune response to Francisella tularensis LVS by administration of anticytokine antibodies. Infect Immun 60: 84–89. 1729199 10.1128/iai.60.1.84-89.1992 PMC257506

[73] Elkins KL, Rhinehart-Jones T, Nacy CA, Winegar RK, Fortier AH (1993) T-cell-independent resistance to infection and generation of immunity to Francisella tularensis. Infect Immun 61: 823–829. 8432603 10.1128/iai.61.3.823-829.1993 PMC302807

[74] Yee D, Rhinehart-Jones TR, Elkins KL (1996) Loss of either CD4+ or CD8+ T cells does not affect the magnitude of protective immunity to an intracellular pathogen, Francisella tularensis strain LVS. J Immunol 157: 5042–5048. 8943413

[75] Conlan JW, Sjostedt A, North RJ (1994) CD4+ and CD8+ T-cell-dependent and -independent host defense mechanisms can operate to control and resolve primary and secondary Francisella tularensis LVS infection in mice. Infect Immun 62: 5603–5607. 7960142 10.1128/iai.62.12.5603-5607.1994 PMC303308

