# Supplementary figures and images for: IKKβ in Myeloid Cells Controls the Host Response to Lethal and Sublethal Francisella tularensis LVS Infection
Source: PLoS One. 2013 Jan 22;8(1):e54124. doi: 10.1371/journal.pone.0054124 (PMC3551972; doi:10.1371/journal.pone.0054124)

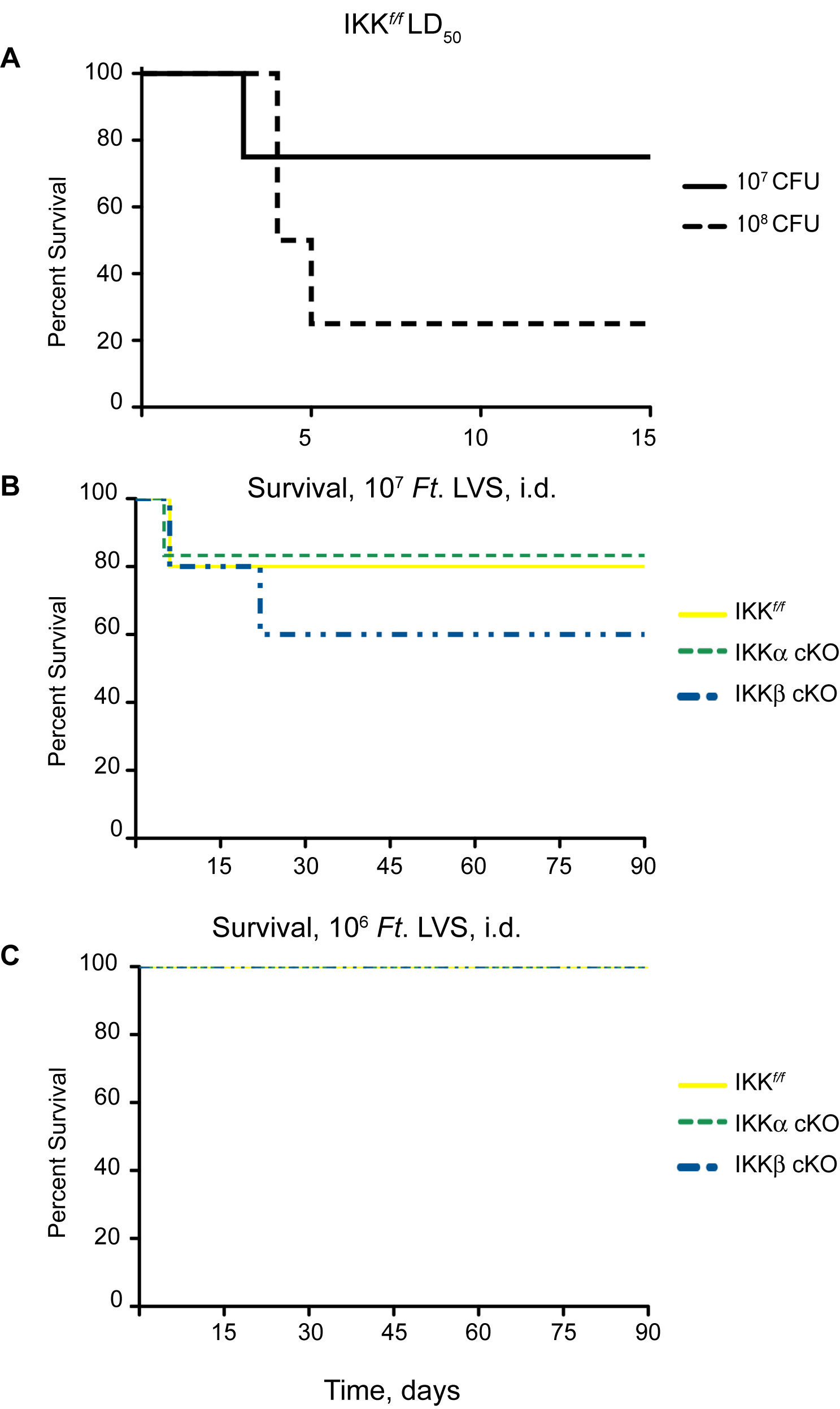

Supplement: Figure S1 — LD50 and sublethal Ft . LVS dose determinations. (A) IKKf/f control mice (n = 5 mice/group) were injected with Ft. LVS i.d. at the indicated doses and analyzed for survival by Kaplan-Meier method in order to determine the median lethal dose. The inoculation dose was confirmed by retroplate assay and the LD50 was estimated at 108 CFU. In panels (B) and (C), IKKf/f, IKKα cKO and IKKβ cKO mice were injected with Ft. LVS i.d at two different sublethal doses: (B) 107 CFU and (C) 106 CFU, and analyzed for long-term survival (n = 5–6 mice/group). (TIF) [file pone.0054124.s001.tif]

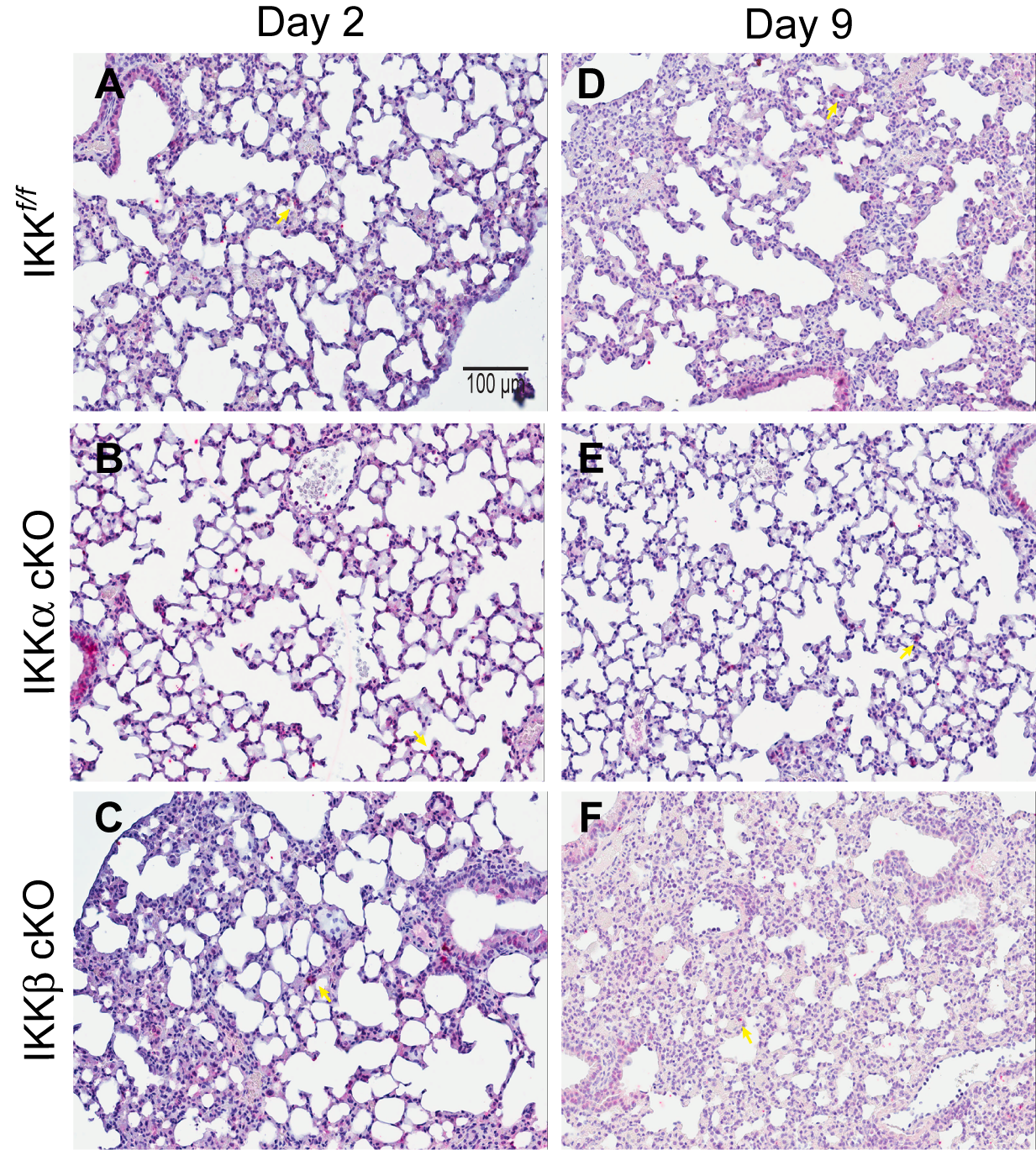

Supplement: Figure S2 — Minimal lung involvement after intradermal Ft . LVS challenge. Lung sections, taken from mice i.d. challenged with a sublethal dose of 106 CFU Ft. LVS, were analyzed by immunohistochemistry for Ft. LVS antigen. (A) and (D) IKKf/f, (B) and (E) IKKα cKO, and (C) and (F) IKKβ cKO are representative lung sections showing low Ft. LVS colonization at days 2 and 9 post infection, respectively. Magnification = 100×, scale bar = 100 µm. (TIF) [file pone.0054124.s002.tif]

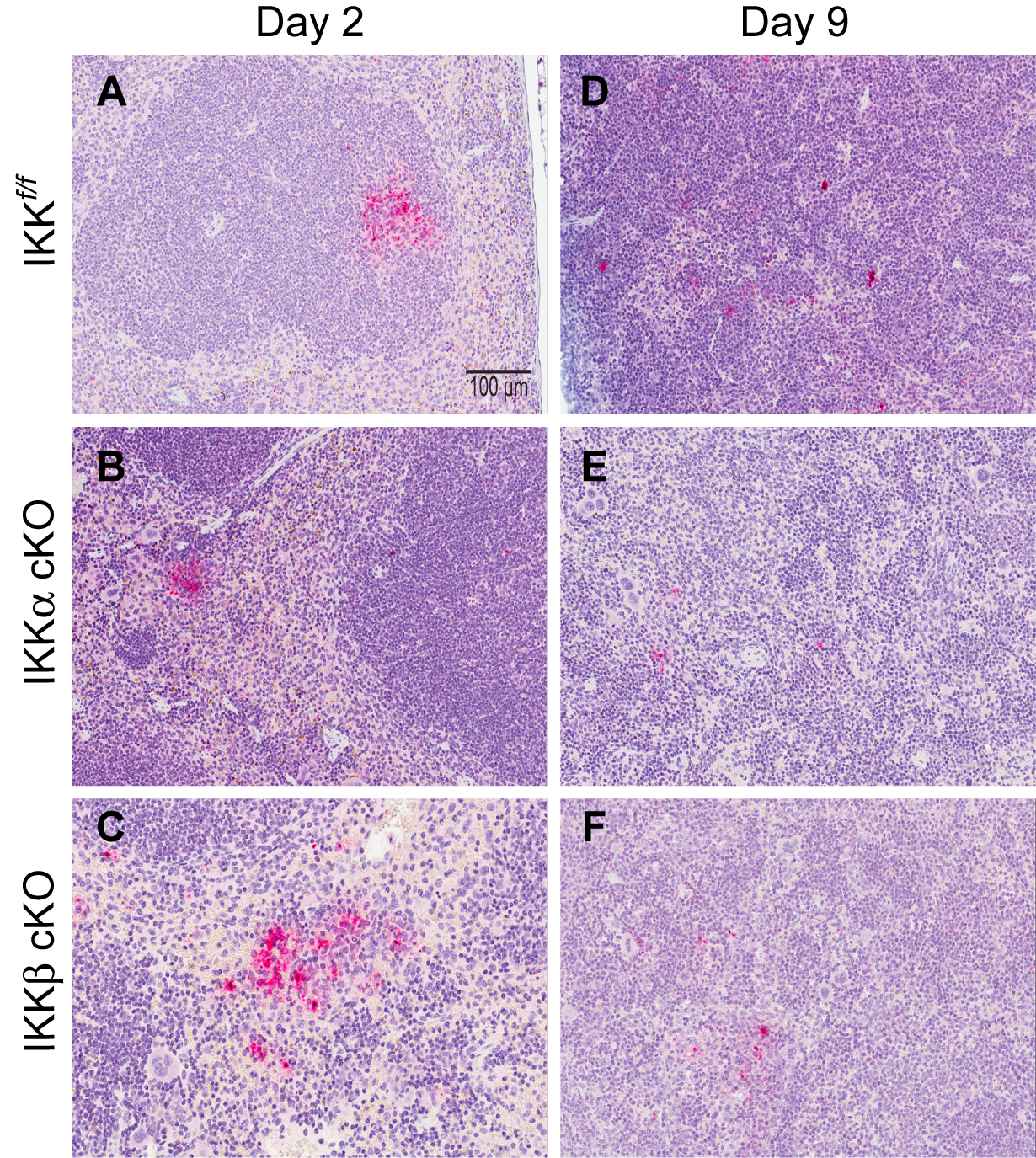

Supplement: Figure S3 — Ft . LVS dissemination in spleen. Spleen sections from mice i.d. challenged with a sublethal dose of 106 CFU Ft. LVS, were analyzed by immunohistochemistry for Ft. LVS antigen. (A) and (D) IKKf/f, (B) and (E) IKKα cKO, and (C) and (F) IKKβ cKO are representative sections of spleens at days 2 and 9 post infection, respectively. Magnification = 100×, scale bar = 100 µm. (TIF) [file pone.0054124.s003.tif]
